# Integration of Social Context vs. Linguistic Reference During Situated Language Processing

**DOI:** 10.3389/fpsyg.2021.547360

**Published:** 2021-08-02

**Authors:** Katja Maquate, Pia Knoeferle

**Affiliations:** ^1^Psycholinguistics, Institute for German Language and Linguistics, Humboldt-Universität zu Berlin, Berlin, Germany; ^2^Berlin School of Mind and Brain, Humboldt-Universität zu Berlin, Berlin, Germany; ^3^Einstein Center for Neurosciences Berlin, Berlin, Germany

**Keywords:** real-time situated language processing, eye-tracking, emotional priming, action depiction, natural facial expressions, schematic faces, social context

## Abstract

Research findings on language comprehension suggest that many kinds of non-linguistic cues can rapidly affect language processing. Extant processing accounts of situated language comprehension model these rapid effects and are only beginning to accommodate the role of non-linguistic emotional, cues. To begin with a detailed characterization of distinct cues and their relative effects, three visual-world eye-tracking experiments assessed the relative importance of two cue types (action depictions vs. emotional facial expressions) as well as the effects of the degree of naturalness of social (facial) cues (smileys vs. natural faces). We predicted to replicate previously reported rapid effects of referentially mediated actions. In addition, we assessed distinct world-language relations. If how a cue is conveyed matters for its effect, then a verb referencing an action depiction should elicit a stronger immediate effect on visual attention and language comprehension than a speaker's emotional facial expression. The latter is mediated non-referentially *via* the emotional connotations of an adverb. The results replicated a pronounced facilitatory effect of action depiction (relative to no action depiction). By contrast, the facilitatory effect of a preceding speaker's emotional face was less pronounced. How the facial emotion was rendered mattered in that the emotional face effect was present with natural faces (Experiment 2) but not with smileys (Experiment 1). Experiment 3 suggests that contrast, i.e., strongly opposing emotional valence information vs. non-opposing valence information, might matter for the directionality of this effect. These results are the first step toward a more principled account of how distinct visual (social) cues modulate language processing, whereby the visual cues that are referenced by language (the depicted action), copresent (the depicted action), and more natural (the natural emotional prime face) tend to exert more pronounced effects.

## Introduction

Monitoring people's gaze behavior in a visual context provides a unique opportunity for examining the incremental integration of visual and linguistic information (Tanenhaus et al., 1995). During sentence comprehension, non-linguistic visual information can rapidly guide visual attention in adults (e.g., Sedivy et al., 1999; Spivey et al., 2002; Chambers et al., 2004; Knoeferle et al., 2005). Crucially, non-linguistic information can also facilitate real-time language processing of canonical and non-canonical grammatical sentences (e.g., Knoeferle et al., 2005; Carminati and Knoeferle, 2013). Social cues, such as, for example, a speaker's emotional facial expression (Carminati and Knoeferle, 2013), a speaker's gaze shift (Kreysa et al., 2018), or the speaker's voice information (Van Berkum et al., 2008), can elicit expectations on the part of the listener (just like other non-linguistic cues), and these can in turn influence the processing of upcoming linguistic information. However, existing research has focused mostly on assessing how object- and action-related visual information influences spoken language comprehension. By contrast, little is known about the effect of social (visual) cues (e.g., an emotional facial expression) on real-time sentence comprehension and which degree of naturalness (and corresponding degree of detail) is needed for comprehenders to exploit them. Additionally, we do not know how far and to which extent (schematic vs. natural) facial emotions and action events relative to one another modulate visual attention and language comprehension. Examining these open issues can further clarify our understanding of the integration of distinct visual cues into language processing. This clarification can in turn help us to refine models of real-time language processing, taking the visual and social context into account.

In most serial language comprehension accounts (e.g., Frazier and Fodor, 1979; Friederici, 2002), contextual representations are depleted and come into play very late during language processing. Parallel interactive theories, by contrast, do not restrict the interaction of information (see also Anderson et al., 2011) and emphasize a rapid interplay between syntactic and non-syntactic representations (e.g., MacDonald et al., 1994; Trueswell and Tanenhaus, 1994). Yet, these constraint-based approaches neither feature interpretation building processes nor non-linguistic representations (see also Novick et al., 2008).

Real-time language processing accounts have largely focused on the integration of visual cues, such as depicted actions and objects, that are referenced by the linguistic input. For instance, the coordinated interplay account (CIA; Knoeferle and Crocker, 2006, 2007) comprises three processing steps. These steps are temporally dependent and can overlap or occur in parallel. In the first step, the linguistic input is interpreted incrementally on the basis of existing knowledge. In the second step, expectations and representations are built and guide attention to relevant information in working memory or a visual scene, e.g., depicting actions and objects. In the final step, previously built interpretations and expectations are reconciled with the scene/working memory representations.

By contrast, we know less about the extent to which further visual cues—that are non-referentially linked to language—impact comprehension [but see, e.g., Guerra and Knoeferle (2014, 2017, 2018) on enriching the CIA with non-referential conceptual co-indexing mechanisms; Altmann and Kamide (1999), Huettig and Altmann (2005), and Altmann and Trafton (2002) for non-referential world knowledge effects on language processing]. Consider, for instance, a speaker's smile that a listener might (or might not) relate to the valence of words in a sentence. Indeed, recent work (Münster and Knoeferle, 2018) has started to extend situated language processing accounts with the biological and experiential properties of the comprehender, as well as with social contextual (visual) information that is non-referential [the (social) CIA (sCIA), see Münster and Knoeferle (2018) also for a more detailed review on how/whether different processing accounts deal with non-linguistic social representations; see also Van Berkum (2018, 2019)]. However, to more fully accommodate how distinct cues contribute toward human language processing, empirical research and these accounts must consider not only the effects of individual cues but also how the effects of (distinct) visual cues measure up against one another. The present research compares the effects of action event depiction with those of (natural vs. schematic) emotional facial expressions (for which the link between the visual and linguistic input is not referential and arguably subtler).

### World-Language Relations in Sentence Comprehension: Referential vs. Non-referential Cues

#### Objects and Actions as Referential Cues

Adults can rapidly use information about objects and depicted action events for disambiguating structurally ambiguous sentences when these cues are referenced and made available by (words in) the utterance. For instance, *kick* refers to a kicking event and makes available the kicking action and knowledge of plausible associated agents such as soccer players. *On the towel* can refer to a location and make available the referent situated at that location. In a real-world study, adults inspected an apple, an apple on a towel, an empty towel, and a box and listened to sentences like *Put the apple on the towel in the box*. Before listeners heard *in the box*, they preferred to interpret *on the towel* as a destination for the apple. However, in a context with two apples, the need to distinguish between them guided participants toward resolving *on the towel* as a modifier of *the apple*, interpreting it as its location: participants quickly looked at the apple on the towel during *on the towel* (location), and not at the empty towel (as a destination) to which the apple could be moved (Tanenhaus et al., 1995; Spivey et al., 2002).

In addition to noun-object relations, adults can use other referential cues, such as verb-mediated depicted action events, to facilitate role assignments, and thus the processing of canonical subject-verb-object (SVO) and noncanonical object-verb-subject (OVS) German sentences [both word orders are grammatical in German but OVS is non-canonical (Knoeferle et al., 2005)]. In a visual-world eye-tracking study, participants inspected clipart scenes depicting a princess as washing a pirate and as being painted by a fencer. The spoken sentence played during scene inspection was initially ambiguous and either related to the princess-washes-pirate event (in SVO order) or the princess-is-painted-by-fencer event (in OVS word order). Shortly after the verb had modulated one of the two depicted actions, participants either visually anticipated the associated pirate (if they had heard *washes*) or the fencer (if they had heard *paints*). From the anticipation of the action's patient (the pirate in SVO sentences) or agent (the fencer in OVS sentences), the authors deduced that listeners had assigned a thematic role to the initially role ambiguous noun phrase *the princess*. Thus, comprehenders can rapidly exploit referential cues (*on the towel* identifying a location; *paints* referencing a painting action and mediating its associated agent) for language processing and the assignment of thematic roles.

#### Non-referential (Visual) Social Cues: Facial Emotions

Would comprehenders also benefit from non-referential visual cues? By “non-referential” (visual) cues, we mean the (visual) information that listeners might associate with language but that is not referentially mediated. The listener has to infer and interpret the relationship between the non-linguistic (visual) cue and the linguistic input in a non-referential way (i.e., hearing *Nice to meet you!* and seeing someone smile), rather than identifying the referential link between a visual cue and a mediating linguistic expression (i.e., hearing *kick* and seeing someone kicking something). A non-referential (visual) cue, such as an emotional facial expression, provides additional non-linguistic information, which could be exploited in order to facilitate linguistic processing and interpretation although it is an interesting issue whether comprehenders can exploit it to the same extent given the non-referential link with language.

Human faces, despite sharing general features, differ greatly in their detailed features (Grelotti et al., 2002). Yet, most people can effortlessly discriminate faces based on those detailed features, making us experts in face recognition (Diamond and Carey, 1986). Becoming an expert in the recognition and processing of faces allows us to interact and communicate with each other (Grelotti et al., 2002). Building this expertise already starts in the earliest moments of life: even newborns, only minutes after birth already attend to faces more than to non-face-like stimuli (Johnson et al., 1991; Mondloch et al., 1999). During communication, we use our face to (consciously or unconsciously) convey a nonverbal message alongside our verbal message. In turn, the listener interprets our facial expression and tries to integrate it into the unfolding interpretation to correctly understand and interpret it or even to facilitate sentence processing (Carminati and Knoeferle, 2013). Emotional priming studies, for instance, show that the valenced positive and negative primes can facilitate and/or speed up the processing and recognition of emotionally congruent subsequent targets [see, e.g., Hermans et al. (1994) and Lamy et al. (2008)].

For example, in a reaction time experiment, Aguado et al. (2007) used faces as primes and words as targets. Participants first saw a positive or a negative prime face followed by either a positive or negative target word or a question mark. If the target word appeared, participants had to judge the valence of the word. If the question mark appeared, the task was to detect the gender of the previously seen positive or negative prime face. Participants did not know in advance whether they had to detect the gender of the face or judge the valence of the word, rendering the task unpredictable. The results were in line with classic priming effects: reaction times were shorter for valence-congruent (vs. incongruent) face-word trials.

Crucially, social visual cues, such as emotional faces, can also affect sentence interpretation. In one study (Münster et al., 2014; Carminati and Knoeferle, 2016), participants inspected a video of a human emotional facial expression. After this speaker's prime face, a new scene appeared showing two event photographs and participants heard a (positively or negatively) valenced sentence related to one of these photographs. The issue was whether a match (vs. mismatch) in the valence of a preceding speaker's face and the valence of the ensuing sentence would boost participants' visual attention to the valence-matching photograph and thus facilitate their sentence comprehension. To experience facilitation, participants had to link the (e.g., positive) valence of the preceding face to the (positive) valence of the ensuing spoken sentence, resulting in a boost of visual attention to a related event photograph. Thus, both links between language and the facial expression were non-referential and the temporal contiguity of the visual cue was less (preceding the target utterance) than for the visual cues examined in a few previous studies [e.g., the action depictions were copresent as comprehenders listened to the utterance in Knoeferle et al. (2005); see Spivey and Geng (2001) and Altmann (2004) on effects in the blank screen paradigm, in which a stimulus sentence is heard after a visual scene had been inspected and removed from the screen; eye movements in the blank screen were measured in response to the sentence].

In spite of these more tenuous world-language links, having seen a smiling/sad speaker face facilitated participants' visual attention and processing of emotionally valenced (positive/negative) canonical SVO sentences (Münster et al., 2014; Carminati and Knoeferle, 2016). The emotional facial expressions were integrated incrementally with the linguistic input and modulated its processing online, again in the absence of referential links. Interestingly, similar effects emerged for static emotional facial expressions (Carminati and Knoeferle, 2013). These findings suggested for the first time that (static and dynamic) facial expressions—like actions—can incrementally modulate adults' processing of emotional sentences. The emotion effects emerged despite the differences in how language conveyed these cues (valence associations vs. verb-action reference) and despite the fact that the speaker's face was not present during comprehension (and thus arguably less accessible). To which extent these findings extend to the processing of other, in particular difficult-to-process non-canonical sentences (e.g., German object-initial sentences) is, however, an open issue.

It is also unclear to which extent the portrayal of emotions (as dynamic human faces or as schematic smileys) matters for emotion effects on language processing. Considering emotional facial expressions, most of the time we interact with other human beings and easily attribute mental states, beliefs, and feelings to our interaction partners, based on our own mental states (“Theory of Mind;” Premack and Woodruff, 1978). We are thus experienced in our interaction with natural human emotional faces. However, research on emotional face recognition has also used computer-generated schematic faces and the evidence suggests that the latter are recognized as well as natural faces (e.g., Öhman et al., 2001; Chang, 2006; Ruffman et al., 2009). ERP (Event-Related Potential) and behavioral research (Schindler et al., 2017; Kendall, 2019; Zhao et al., 2019) suggests that (emotional) cartoon faces are recognized faster and might be analyzed more on a structural level compared with natural human faces (as indexed by shorter RT (Reaction Times), briefer N170 ERP latencies, and larger N170 amplitudes). By contrast, natural (vs. cartoon) faces are processed more holistically and require more attentional resources during later processing stages [as indexed by larger late positive potential (LPP) amplitudes].

Whether schematic (vs. natural human) facial emotions would yield comparable effects also for real-time visual attention and language processing is, by contrast, an open issue. Is a schematic expression sufficient (e.g., as in smileys, where emotion is stripped down to its bare essential, perhaps rendering valence salient), or do emotional priming effects on online sentence comprehension emerge only following more realistic, detailed, and natural emotional faces?

#### Toward Differentiating World-Language Relations

The present research compared the effects of two distinct cues (referentially mediated actions and their associated agents with non-referential facial expressions) within a single study, and in addition manipulated the degree of naturalness of the facial expression on incremental sentence processing within a single study, and in addition manipulated the degree of naturalness of the facial expression.

Extant research has begun to compare the influence of referential (object depiction) and non-referential (speaker gaze shift) cues on sentence processing (Kreysa et al., 2014, 2018). In a visual-world eye-tracking study, participants inspected the videos of a speaker uttering German sentences about two virtual characters (translated, e.g., *The waiter congratulates the millionaire in the afternoon* with a Second Life display showing a saxophonist, waiter, and millionaire). The action was (vs. was not) depicted and the speaker either shifted gaze between the characters referred to in the sentence or was obscured, yielding four conditions (neither gaze nor action was present; only either gaze or the action was present; and both of these cues were present). Both cues appeared simultaneously, just after the onset of the verb (the speaker shifted gaze to the millionaire and the action tool appeared between the waiter and the millionaire). Listeners used both cues to anticipate the upcoming patient of the sentence (the millionaire) before its mention. The speaker gaze cue enabled anticipation reliably earlier than the action cue but only when the action was used non-deictically (Kreysa et al., 2018; Experiment 2). When both action depiction and gaze were used deictically, their effects on visual attention and comprehension were comparable. Two cues did not seem to be more helpful than one when the action was used non-deictically.

Due to the diverse nature of the different kinds of cues, we do not yet know if distinct language-world relations ease utterance interpretation to the same extent and in a similar fashion. It could be that the speaker gaze is so effective because it is dynamic and present during comprehension, and the dynamic motion captures and guides listeners' attention. But emotional facial expressions might be as effective as gaze, permitting rapid anticipation: seeing, for example, our interlocutor smile likewise sets up expectations as to what might come next. These might be expectations about a matching emotionally positive surrounding situation. Moreover, it likely fosters the expectation that the upcoming utterance is also positive in emotional valence. Both speaker gaze and a speaker's emotional facial expression raise expectations; we can link these cues to linguistic material in an utterance matching these expectations and could direct attention to relevant parts in a visual scene.

Carminati and Knoeferle (2013) provide some evidence for rapid effects of a preceding emotional speaker's face for the subsequent processing of at least German subject-initial sentences (and this despite the fact that the speaker's face was not co-present during comprehension). Seeing someone smile and hearing an emotionally positive linguistic expression, such as *happy*, does not foster a referential link (as between an action verb and a perceived action): the hearer first has to recognize and interpret the emotional facial expression, likely resulting in the activation of a representation of the concept of happiness. This might set up expectations regarding the emotionality of the situation between a speaker and a hearer. When the emotionally positive adverb *happily* is encountered, this concept has to be linked in a non-referential way to the encountered linguistic expression. Then, attention can be directed to the visual input in a scene, e.g., seeing another person, such as the agent of an action, smile. Hence, even though there is a link between a speaker's smile and an associated linguistic expression, this link is not referential and arguably more complex than a referential link. Had the linguistic expression been *cheerful* or *friendly* instead of *happily*, a very similar or even identical link to the link between a smile and the word *happily* could have been established.

Hearing an action verb (e.g., *kick*), on the other hand, directs the hearer's attention *via* a referential link to a depiction of the heard action verb (e.g., a man standing on a field who is stretching out one leg in a kicking action). The world-language link is referential because no intermediate processing steps, such as forming non-referential conceptual representations, interpreting these representations in the present situation, and relating them to the perceived action, have to be performed.

As a few previous studies investigating referential and non-referential world-language relations suggest (see Sections Objects and Actions as Referential Cues– Toward Differentiating World-Language Relations), expectations are set up and attention is directed *via* these links to relevant parts in a visual scene when both referential and non-referential links can be established. Perhaps then we will see no difference in the effects of emotional facial cues and actions? Alternatively, the actions are referential (and present during comprehension), and could hence elicit stronger effects than facial cues that are (non-referentially) related to the emotional valence of, for instance, sentential adverbs.

Please note that our aim was neither to test verb cues vs. emotional cues nor to generalize across all referential vs. non-referential cues. Instead, the goal of the present research was to determine how emotional facial expressions (as one specific example of non-referential cues) and depicted action events (as one specific example of referential cues) affect online sentence comprehension. To what extent and in what way do these cues interact with each other, and to what extent does the naturalness of the cue (the emotional facial expression) matter? We acknowledge that other referential and non-referential cues might differ from emotional facial expressions and depicted actions in the way and the degree in which they link to language. Yet, as there is no prior research in this domain (that we are aware of), we chose emotional facial expressions and depicted actions as cues to maximize the difference between referential and non-referential relations and because these cues have already been shown to affect language processing on their own (cf., Knoeferle et al., 2005; Carminati and Knoeferle, 2013). Examining these issues will provide further insights into the relative effect of distinct kinds of cues on language processing. Models of language processing, such as the (social) CIA (CIA; Knoeferle and Crocker, 2007; sCIA: Münster and Knoeferle, 2018), are underspecified regarding the relative integration of different kinds of extralinguistic cues into language processing since empirical evidence is lacking. They are also underspecified regarding effect-differences for natural compared with schematic cues (e.g., facial expressions). The results of the present research can thus inform their extension.

Three visual-world eye-tracking studies compared the effects of action event cues with those of a speaker's emotional facial expression as a prime (Experiments 1 and 2) and manipulated naturalness of the facial expressions across experiments (Experiments 1–3). To further examine whether facilitative effects of the emotion cues (Carminati and Knoeferle, 2013) extend to other sentence structures and processes of the assignment of thematic roles, we employed noncanonical (but grammatical) German OVS sentences. We know that the actions facilitate OVS sentence comprehension and examine whether the effects of emotional facial stimuli previously only attested for SVO sentences would generalize and be comparable in their effects. Experiment 1 examined the effects of schematic facial expressions and Experiment 2 of natural facial expressions on the assignment of thematic roles during the comprehension of spoken German OVS sentences. Experiment 3 further investigated the effect of natural facial expressions in the absence of depicted action events and set a stronger focus on language processing situated in a more salient emotionally valenced environment.

## Experiment 1

### Methods

#### Participants

40 students of University of Bielefeld between 18 and 30 years (14 male, mean age: 24, SD age: 3.09), all native speakers of German, took part in the experiment. Participants were tested in the eye-tracking laboratory of Bielefeld University. Sample size was set to 40 to ensure comparability with a related study with children as participants. Each participant received 4 Euro for participation and gave written informed consent. The university's ethics board approved the study (Vote 2013-007). The experimental session took about 30 min.

#### Materials

The design crossed *emotional prime* (prime valence congruous vs. incongruous with the sentence) with *action* (present vs. absent). We realized the first factor of the design *via* prime images (a yellow smiley vs. a red star) constructed by using commercially available software (see Table 1). The smiley changed dynamically from a light and subtle to a broad smile. The red star was static and had no facial features. The smiley matched the target sentences in valence (see Table 1) while the red star was incongruous in that it conveyed no emotional valence *via* facial features. We avoided negative emotional primes for consistency with a planned child study.

**Table 1:** Experimental conditions for Experiment 1.

**Condition**	**Prime**	**Action**	**Sentence**
A	Happy yellow smiley, congruent prime	Action depicted	*Den Marienkäfer kitzelt vergnügt der Kater* [“The ladybug (accusative object, patient) tickles happily the cat (nominative subject, agent)”]
	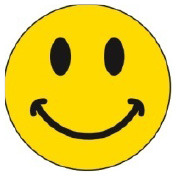	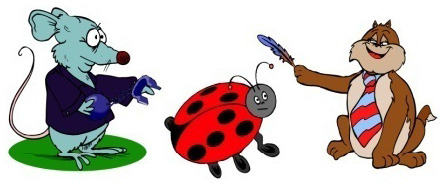	
B	Happy yellow smiley, congruent prime	No action depicted	
	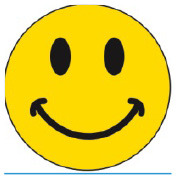	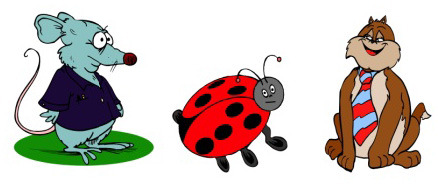	
C	Red star, incongruent prime	Action depicted	
	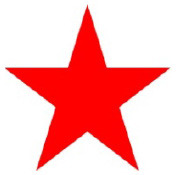	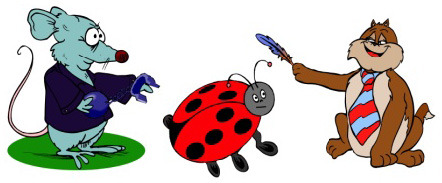	
D	Red star, incongruent prime	No action depicted	
	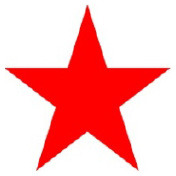	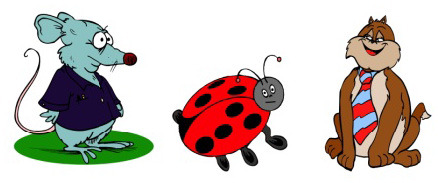	

The second independent factor was realized *via* the target scenes (*N* = 16). These were created by using Adobe Illustrator and commercially available clipart. Most of the clipart characters were animals, some humans (i.e., three human target agents, one human patient/middle character). Each scene consisted of three clipart characters and either depicted actions (see Table 1, A/C) or not (Table 1, B/D). The middle character was always the patient of the action performed by the outer characters. Only one of the actions performed by the outer characters was mentioned in the target sentence (see below) and its agent was the target agent; the other outer character performed an action, which is not mentioned in the sentence (competitor). The target agent (only) portrayed a happy facial expression (matching in valence with the prime smiley). The patient had a neutral and the competitor had a slightly negative facial expression. To counterbalance the position of the agent and competitor, we created a mirrored version of each experimental target scene. In one version of a target scene, the agent was thus on the right-hand side of the picture and in the other it was on the left.

For the experimental target sentences, we constructed 16 unambiguous noncanonical OVS sentences in German [e.g., *Den Marienkäfer*_NP1[masculine accusative case, patient]_ *kitzelt*_action verb_ *vergnügt*_adverb [positive emotional valence]_ *der Kater*_NP2 [nominative case, target agent]_, transl.: “The ladybug (acc. obj., patient) tickles happily the cat (nom. subj, agent)”, see Supplementary Material and Appendix in Münster (2016) for materials]. A female speaker (PK) recorded the sentences with neutral intonation and at a slow but natural sounding speed. Word region onsets and offsets were marked for later analyses. In addition to the experimental target sentences, the same speaker recorded comprehension questions in the active or passive voice, asking either for the agent or the patient of the sentence (e.g., “Who is doing [previously named action] to [previously named patient]” and passive questions in the fashion “Who is being [previously named action]?”).

In addition to the experimental items, we also constructed filler items (*N* = 28). These comprised filler sentences in either an unambiguously case-marked SVO (*N* = 24) sentence structure or an unambiguously case-marked OVS (*N* = 4) structure, recorded by PK. Some filler sentences had neutral verbs and adverbs (*N* = 12, thereof the four OVS sentences) and some were positively valenced (16 SVO sentences). The corresponding 28 filler pictures consisted of clipart animals and humans. Some always depicted three (*N* = 12) and others two characters (*N* = 16). The filler characters were positioned such that the interacting characters faced each other or looked away from one another; such that the agent faced the competitor character; or such that they faced the participant. This was done to prevent participants from developing a strategy as to who will be interacting with whom. Characters had a positive facial expression when the sentence was positive (*N* = 16). When the sentence was neutral (*N* = 12), their facial expressions were also neutral or slightly negative. Half of the filler scenes depicted the action mentioned in the sentence (*N* = 14) while the other half depicted no actions (*N* = 14).

#### Pretests

We pretested the characters and actions to ensure that participants can recognize them. Moreover, we tested the valence of the emotional adverbs. Since we planned to conduct future child language studies using the same materials, we pretested the stimuli with a sample of 4–5-year-old children (*N* = 20, mean age: 4.8). About 10 children were asked in German to point to the agent and patient characters and the actions of the experimental scenes when an experimenter named them (transl.: *Who is the cat? Who is tickling the ladybug here?*). Character naming trials (presented in the no-action condition) and action naming trials (presented in the depicted action condition) were blocked. In this way, the characters were named before participants identified the character performing the action. The children identified the characters (96.9%) and the actions (88.5%) accurately. Ten additional children were asked to identify the happily acting (target) agent (transl.: e.g., *Who tickles happily the girl?*). For this second test, the target agent and the competitor character performed the same actions (i.e., unlike in our experimental pictures) but only the target agent smiled. Experimental scenes were mixed with filler pictures and sentences, which conveyed a negative or neutral valence. In 89.38% of the cases, children reliably identified the happy agent and thus successfully linked the positive adverb to the happy target agent.

In summary, a 2 (congruent smiley prime vs. incongruent red star prime) × 2 (action depiction vs. no action) design yielded four conditions (Table 1). The depiction of the prime and the action described by the sentence varied across conditions while the sentence was identical. We created eight lists such that each participant encountered all conditions but each sentence in only one of the four conditions (Table 1). These four lists were doubled to accommodate the mirrored character scenes (see Section Materials) yielding eight lists. Moreover, in each list, half of all comprehension questions were asked in the active and half in the passive voice, and each experimental item was followed equally often by active and passive questions. Each list contained all of the filler trials and was pseudorandomized for each participant. Two critical items never followed another.

### Hypotheses

#### Accuracy

Adults can use case marking to reliably identify OVS word order (e.g., Kamide et al., 2003a, Experiment 3; Kamide et al., 2003b, Knoeferle et al., 2008). Thus, at sentence end, we did not predict significant effects of the prime and action manipulation on accuracy in the comprehension questions.

#### Eye Movements

Eye movements, by contrast, provide insights into real-time comprehension in the four conditions. We expected condition differences to the extent that the different cues elicit distinct effects on visual attention and sentence comprehension. If two cues are better than one, then participants should look more and earlier toward the target agent (vs. the competitor), signaling anticipation of the correct role filler when both depicted action and prime smiley are present (vs. the single cue conditions). As the verb refers to the action, guiding the listeners' attention to the action and its associated agent, we predicted this cue to have a stronger effect than the smiley prime, which provides a non-referential link to the target agent. As a result, participants should look more toward the target agent (than the competitor) when only the action than when only the smiley was available. When the actions and smiley were absent, we predicted no clear fixation differences between the target agent and the competitor.

### Procedure

**Figure 1:**
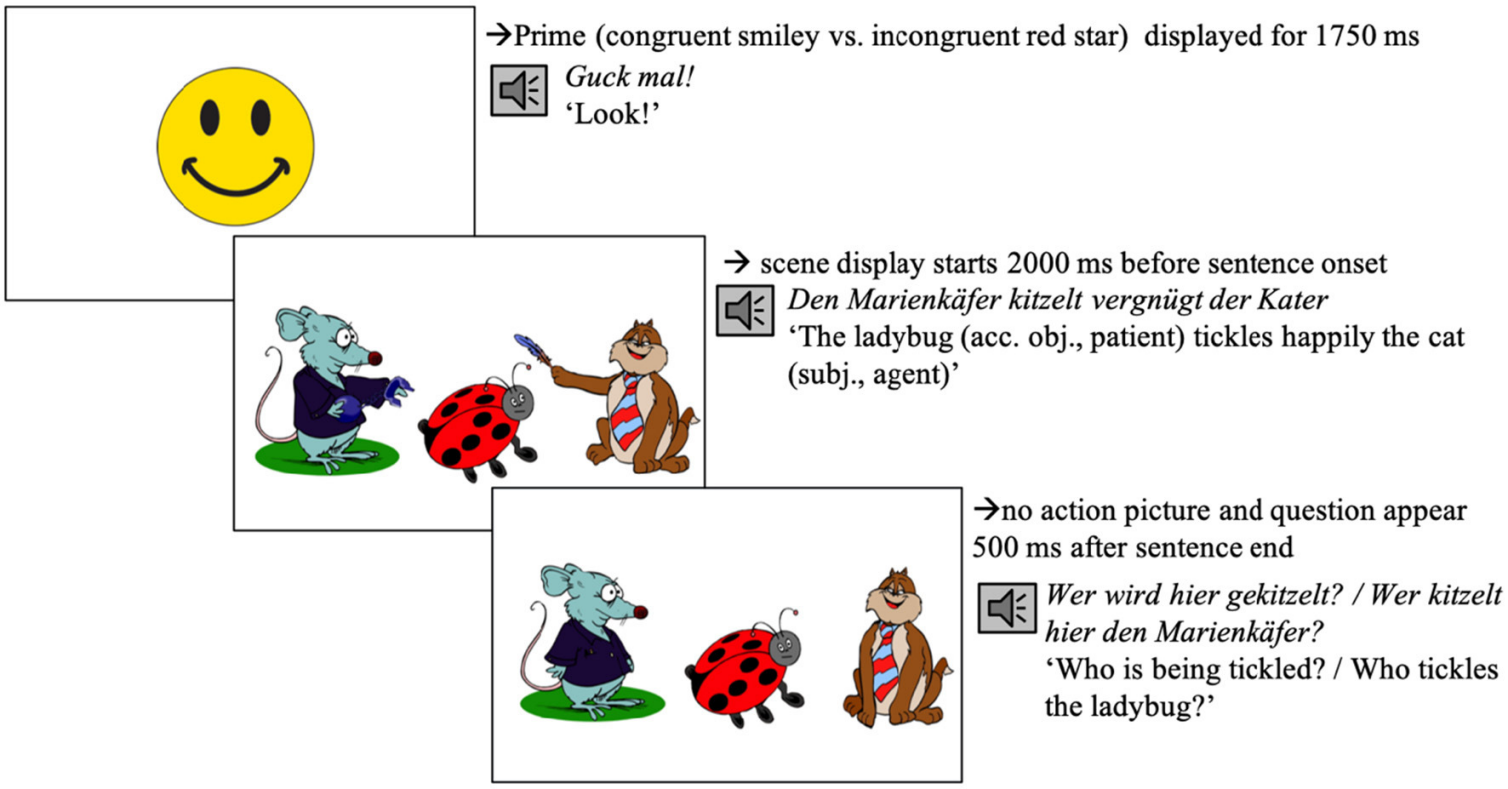
Procedure of an experimental trial in condition A (see Table 1), both cues present (Experiment 1). Note on image clarification: the target agent (i.e., the cat) is tickling the ladybug using a feather; the competitor (i.e., the rat) is arresting the ladybug using handcuffs.

All participants first read a participant information sheet and gave written informed consent. They were seated in front of the Eye tracker (Eye-Link 1000 Eye tracker, SR Research, Ontario, Canada) in the remote setup and asked to read the on-screen instructions. These instructions informed them that they would see a series of scene-sentence pairs. They were asked to concentrate on the scenes and to listen closely to the sentences. They were informed that they would have to answer a question about what they saw and heard after each trial. The experimental session started with a manual five-point calibration and validation procedure. Calibration and validation were repeated when necessary during the experiment. After successful calibration and validation, participants completed four practice trials. The experimenter advanced each trial manually after participants successfully fixated the black dot. The fixation dot was followed by the presentation of the prime smiley video (duration: 1,750 ms, changing from a slight to a full smile after 250 ms), which was accompanied by the phrase *Guck mal!* (*Look)*. That phrase served to focus participants' attention and was inserted with a view of planned developmental studies. After the prime, the target scene was previewed for 2,000 ms (Figure 1) after which the sentence started. 500 ms after the end of the sentence, the actions (if depicted) were removed from the scene and participants heard a comprehension question while looking at the no-action scene (Figure 1). Participants had no time limit and responded orally. After the participant had responded^1^, the experimenter wrote the answer down and started the next trial. At the end of the experiment, participants were debriefed: They were asked to report what they thought the experiment was about; whether they noticed anything odd and/or any regularities; and whether they developed any strategies during the experiment.

#### Exclusion Criteria

If a participant had guessed the purpose of the experiment (nearly) correctly (i.e., “I think the experiment investigates how depicted actions and emotional facial expressions influence language processing”), the participant's data would have been excluded from the data set. This was, however, not the case for any of the participants. Additionally, the fixation data from all experiments was first manually inspected to see if all participants executed fixations to the prime and target scenes in a natural way. If, for instance, a participant had always fixated the middle of the screen or only always the character on the right side of the screen, this participant would have been excluded. Since fixation patterns of all participants seemed to indicate natural fixations of the screens, no participant was excluded.

### Analysis

#### Eye Movements

The eye-movement analyses included the data from all experimental trials (correctly and incorrectly answered), since the accuracy for determining thematic roles was at 96%. We divided the target scenes into a target agent and a competitor character and analyzed the real-time data from the target scene presentation onset until 500 ms after sentence offset. The item sentences were divided into individual analysis regions (see Table 1): NP1 (i.e., the patient is named), verb, adverb, a combined verb-adverb region to capture spillover effects, and an NP2 (i.e., target agent is named) region. Additionally, we computed a “long region,” spanning from NP1 onset until sentence offset plus 500 ms. Our critical time regions were the verb and adverb, since in the depicted action condition, the verb denotes the first region in which the agent of the sentence can unambiguously be determined. The adverb is the first region, which explicitly conveys linguistic emotional valence information. In the no-action condition, it denotes the first region in which sentence valence can be integrated with the emotional prime to anticipate the target agent based on its emotionally matching facial expression.

To exclude any prior preference in looks toward the agent vs. the competitor, we also analyzed the fixations during the NP1 region. To capture the effects of prime and action during the naming of the target agent, we also analyzed the NP2 + 500 ms region. Finally, extended effects across the sentence were analyzed by using the long region.

Fixations were measured by using the natural logarithm (based on the constant *e*) of the ratio of the probability of looking at the target agent over the probability of looking at the competitor character [*ln*(*p*(agent)/*p*(competitor))]. The log ratio is symmetrical around zero. This means, a positive value indicates a preference to look at the target agent over the competitor. A negative log ratio indicates a preference to look at the competitor over the target agent. A value of zero indicates no preference for either of the two characters. Since the log of zero is undefined, we added a constant of 0.1 to account for missing data points regarding fixations to both the agent and the competitor. Hence, this log probability ratio expresses the strength of the visual bias toward the target agent relative to the competitor character. Additionally, it has the advantage that it does not violate the assumptions of independence and homogeneity of variance (Arai et al., 2007).

**Figure 2:**
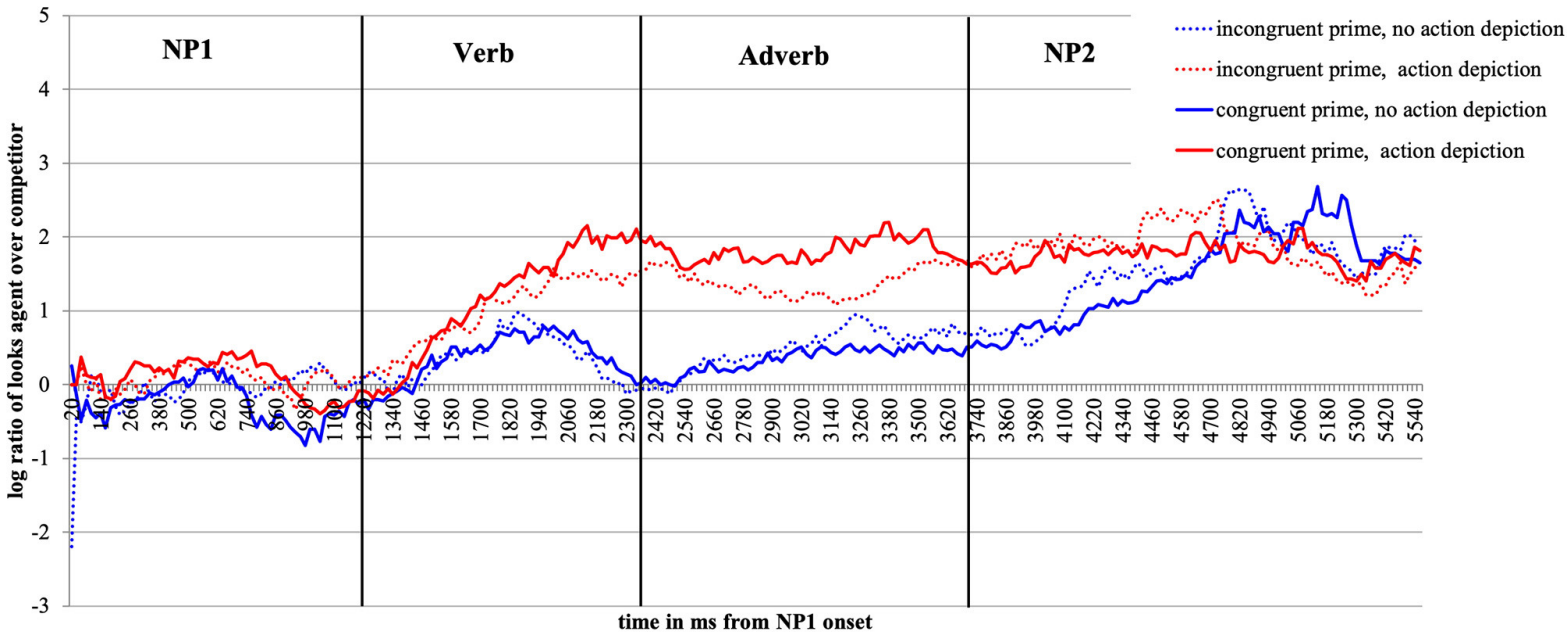
Time-course graph Experiment 1 from NP1 onset until onset of the question display.

For visual presentation, we plot time course graphs as a function of prime and action depiction (Figures 2, **5**) and as a function of prime (**Figure 10**) using the mean log gaze probability ratios calculated on successive 20-ms time slots. For the inferential analyses, the log ratios were subjected to linear mixed-effects models [using *lmer* of the lme4 package of R (Bates et al., 2015b)] with action (no action vs. depicted action), prime (congruent vs. incongruent) as fixed factors, and participants and items as random intercepts. All factors were centered (to avoid collinearity) and sum coded. We included random slopes for action and prime in the participant and item random effect structures and, following (Bates et al., 2015a), we are reporting the results for the best-fitting (most parsimonious) models. The syntax for the best-fitting models for each analysis is reported in footnotes. We obtained the best-fitting models by reducing the random effect structure, starting with the maximal model [log_ratio ~ action^*^prime + (1+action^*^prime | participant) + (1+action^*^prime | item)]. The fixed effect structure of the model was not reduced. We calculated the values of *p* using the *lmerTest* package [Kuznetsova et al. (2017), i.e., Satterthwaite degrees of freedom method (cf., Luke, 2017)].

#### Accuracy Data

Accuracy was computed on the correct and incorrect comprehension-question responses for experimental trials (*N* = 640). To analyze accuracy, we ran generalized linear mixed-effects models in R [R Core Team, 2021; *glmer* function in the *lme4* package (Bates et al., 2015b)]. All models used emotional prime (congruent vs. incongruent) and action depiction (depicted action vs. no action) as fixed factors and subjects and items as random intercepts. Prime and action were included as random slopes into the subject and item random effect structure. Random effect structure selection followed the same procedure as for the eye-tracking data analyses. Question voice (active vs. passive) was used as an additional fixed factor and was likewise a factor in the random slopes. In all models, “family” was set to “binomial” due to the categorical nature of the accuracy scores.

### Results Experiment 1

#### Descriptive Eye-Movement Results

Figure 2 plots the time course of fixations to the target agent relative to the competitor character from the onset of NP1 until the end of the target display in bins of 20 ms. As expected, upon hearing the patient named (NP1), participants show no gaze bias to either the agent or the competitor character. Upon encountering the verb, participants begin to look more at the target agent than competitor, and more so in the action (the red lines) than no action (blue lines) conditions. This effect lasts until the agent is mentioned (middle of NP2).

Focusing now on the contrast between the valence-congruent (smiley) prime and the incongruent (red star) prime, we see no preference in looks toward the agent (vs. competitor) during the verb and adverb^2^ when no action was depicted (the two blue lines do not diverge). However, if an action was present (red lines), the presence of the congruent smiley (vs. the incongruent red star prime) drew subtly more looks toward the agent during the verb and especially the adverb region (the solid vs. the dotted red line, respectively). During the NP2 region (target agent named), the red and the blue lines begin to converge as the agent was named and could thus be discriminated.

#### Inferential Eye-Movement Analyses

**Figure 3:**
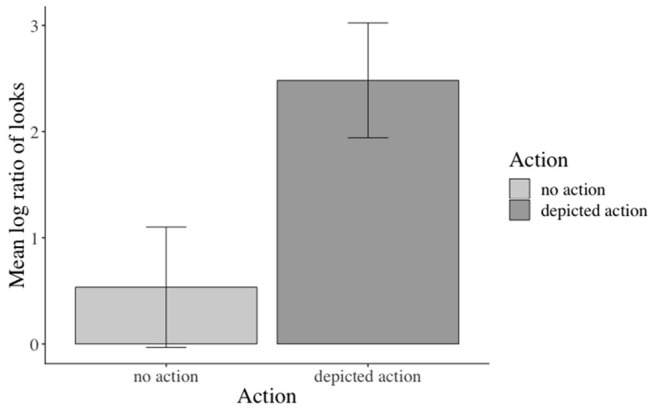
Significant main effect of action in the verb region. Error bars = 95% CIs.

**Figure 4:**
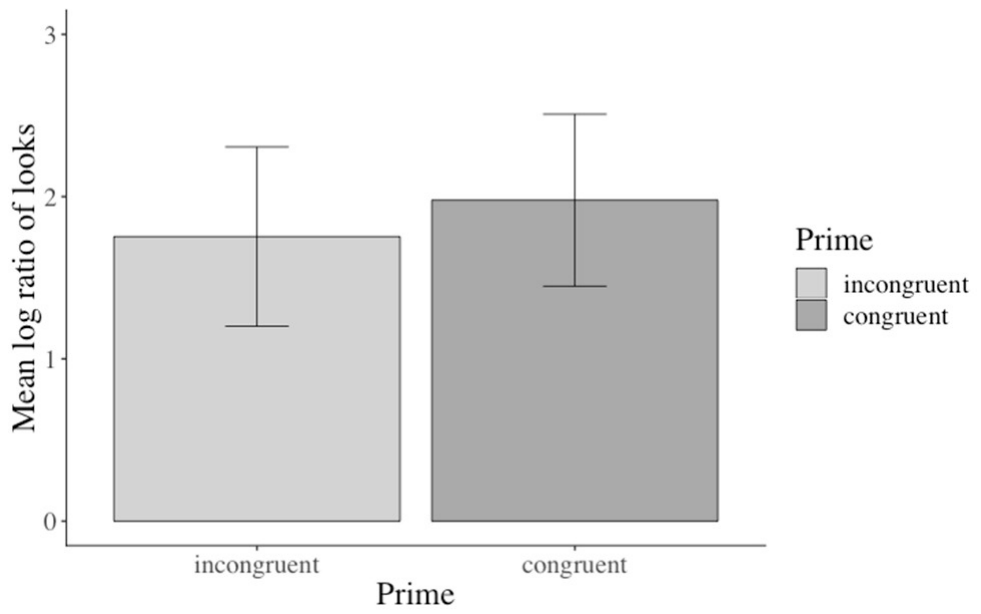
Non-significant effect of emotional congruence in the verb-adverb region. Error bars = 95% CIs.

The inferential analyses revealed significant main effects of action in the verb^3^, adverb^4^, verb-adverb^5^, NP2^6^, and the long region^7^ (e.g., verb region: β: −0.973, SE: 0.141, *df* : 582.000, *t*: −6.888, *p* < 0.001), but not in the NP1^8^ region (see the Supplementary Material for additional model parameters of all analyses). Participants fixated the agent significantly more than the competitor when an action was depicted (vs. no action depiction). Moreover, this effect started as soon as the verb information became available (see Figure 3). No effects of emotional prime emerged (see Figure 4).

#### Accuracy Results

Participants answered 96% of all trials correctly. The analyses yielded no significant effects of the independent factors regardless of whether question voice (active vs. passing) was an additional factor in the model or not^9^.

### Discussion Experiment 1

The eye-tracking pattern corroborated that from the verb onward people made extensive use of the depicted actions for discriminating the target agent of the OVS sentence. By contrast, effects of the emotional prime on sentence interpretation were not reliable. One reason for the absence of clear effects of the emotional smiley on the eye-movement pattern could be that the smiley did not evoke a strong impression of positivity, eliciting accordingly a fairly small (and nonsignificant) priming effect on the processing of the emotionally positive sentence.

Although schematic portrayals of emotions, such as the smiley, might highlight the emotional expression and focus on positivity, its schematic nature could also impede strong emotion effects on language processing: emotional facial expressions are reflections of dynamic psychological and physiological states that vary not only from person to person but also in their degree of positivity or negativity and meaning in context. Recognizing emotion from even natural faces out of context is difficult (Barrett et al., 2007). That difficulty might prevent language users from fully and rapidly exploiting facial expressions during language comprehension, especially when the emotional facial expression is presented as the first stimulus (without prior context) and when it is not referenced by language.

At the same time, recognizing our interlocutors' (facial) emotion during an interaction is vital for building and maintaining social relationships; for nonverbal communication, and moreover for interpreting the other person's feelings (Lamy et al., 2008). Perhaps then, a real human speaker face (in contrast to a schematic depiction) is needed for enabling effects of the facial prime during language comprehension. In fact, emotions from a human face are recognized faster and more accurately and elicit enhanced and prolonged cortical responses when they are presented in a more natural manner (dynamic) compared with static [see, e.g., Harwood et al. (1999) for the identification of emotions from moving and static videotaped and photographic displays; Recio et al. (2011) for ERP evidence]. In addition, viewing a smiley prime but then hearing a human voice may have decreased listeners' integration of the prime face with the speech and its associated valence. In Experiment 2, we accordingly hypothesized that using a video of a human speaker's (facial expression) might lead to stronger priming effects than a schematic smiley.

Another explanation for the weak effects of the emotional prime could be a task bias. Since the comprehension questions focused on thematic role relations, participants may have focused on the actions, boosting their effects on comprehension. This explanation receives support from a study by Hajcak et al. (2006) in which an ERP component (the LPP) often observed in emotion processing was significantly smaller for non-affective than affective judgments of emotionally valenced pictures. In Experiment 2, we complemented the questions about who-did-what-to-whom with an emotion-verification task (see below).

In terms of the performance on the comprehension task, participants' scores were at ceiling in all four conditions. Exploratory analyses revealed that participants answered slightly more passive than active comprehensions questions incorrectly (passive: 3.4% vs. active: 0.6%; difference *n.s*.). The at-ceiling accuracy values especially in the active voice might have concealed off-line effects of the emotional prime and action factors. To explore this possibility, Experiment 2 posed all experiment-trial questions in the passive voice.

## Experiment 2

### Methods

#### Participants

A further 40 students of University of Bielefeld, all native speakers of German with normal or corrected-to-normal vision, between 18 and 30 years (15 male, mean age: 23, SD age: 3.62) took part in the experiment. Participants were tested in the eye-tracking laboratory of Bielefeld University. Sample size was set to 40 to ensure comparability with a related study with children as participants. Participants received 4 Euro for their participation. All gave written informed consent. Ethics approval was given by the university's ethics board (Vote 2013-007).

#### Materials

The sentences and scenes were the same as in Experiment 1. However, the comprehension questions for the 16 experimental sentences in Experiment 2 were always asked in the passive voice. Across all trials, participants heard equally many active and passive comprehension questions. To foreground emotional valence, the experimenter asked the participant to recall the prime speaker's facial expression after four experimental trials (one per condition). These questions were counterbalanced so that across participants they appeared after each item in each experimental condition. In addition, after 12 of the 28 filler trials participants had to identify emotions (“How are they feeling?”): they saw (posttrial) the facial expression of the speaker (always same valence as the prime) next to the face of one of the characters from the previously seen target scene. The same female speaker who recorded the item sentences recorded these questions. In the remaining 16 filler trials, participants were asked the same active question about the agent/patient of the sentence as in Experiment 1.

The emotional prime in Experiment 2 consisted of face videos. In the positive video, a woman changed her facial expression from neutral into a broad smile (video duration: 5,500 ms; change to positive after 1,300 ms). The negative video was constructed in the same way but the woman's face turned from a neutral into a sad expression (see Table 2). We chose the woman's face based on a previous rating study of facial emotion photos [DeCot (2011); unpublished Master's thesis, *N* = 18, mean age: 24.7]. In that study, the woman's happy and sad facial (static) expressions were one of the three most recognizable among 15 faces (large differences between neutral, positive, and negative emotions). We recorded that woman for the videos of the present study^10^.

**Table 2:** Experimental conditions for Experiment 2.

**Condition**	**Prime**	**Action**	**Sentence**
A	Happy dynamic natural facial expression, congruent prime	Action depicted	*Den Marienkäfer kitzelt vergnügt der Kater* [“The ladybug (accusative object, patient) tickles happily the cat (nominative subject, agent)”]
	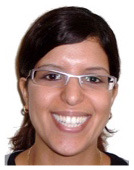	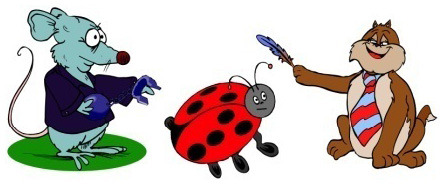	
B	Happy dynamic natural facial expression, congruent prime	No action depicted	
	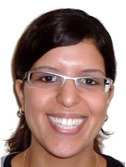	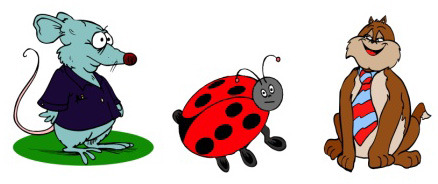	
C	Sad dynamic natural facial expression, incongruent prime	Action depicted	
	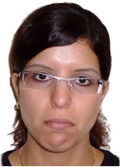	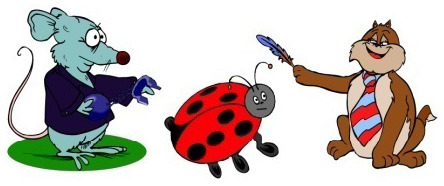	
D	Sad dynamic natural facial expression, incongruent prime	No action depicted	
	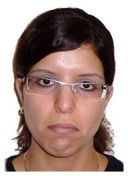	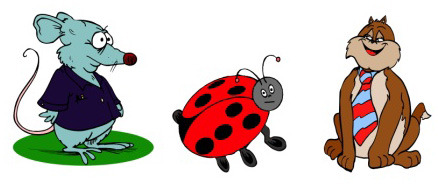	

### Design and Hypotheses

The design was identical to Experiment 1 (2 × 2, action × emotional prime) with the exception that for the prime face, only the emotional expression varied in valence (Table 2). For the accuracy data, we predicted to replicate the results from Experiment 1. However, to the extent that the passive questions in Experiment 2 minimize the potential ceiling effects (see Experiment 1), we expected to see more correct answers when the actions were present (vs. absent), and the emotional prime face was congruent (vs. incongruent). Participants should further correctly answer the recall questions about the emotional valence of the prime face in the experimental trials. For the eye-movement behavior, we also expected to replicate the fixation patterns from Experiment 1. However, given the increased task focus on emotional valence, we predicted stronger effects in Experiment 2 than 1. If both the naturalness of the prime face and the increased task focus on emotions boost agent anticipation, fixations toward the agent (vs. competitor) should be more pronounced in Experiment 2 than 1. We predicted no between experiment differences for the action effects.

### Analyses

The analyses for the eye-movement data were the same as in Experiment 1. Accuracy scores for the face recall task were not analyzed inferentially since they only yielded four data points per participant. Accuracy scores for the questions about how the characters are feeling were also not analyzed as these questions were only asked on filler trials. Analyses of the comprehension question scores were performed similar to Experiment 1 using prime and action as fixed factors. However, since all experimental comprehension questions were in the passive voice, voice was not included in the models.

### Results Experiment 2

#### Descriptive Eye-Movement Results

**Figure 5:**
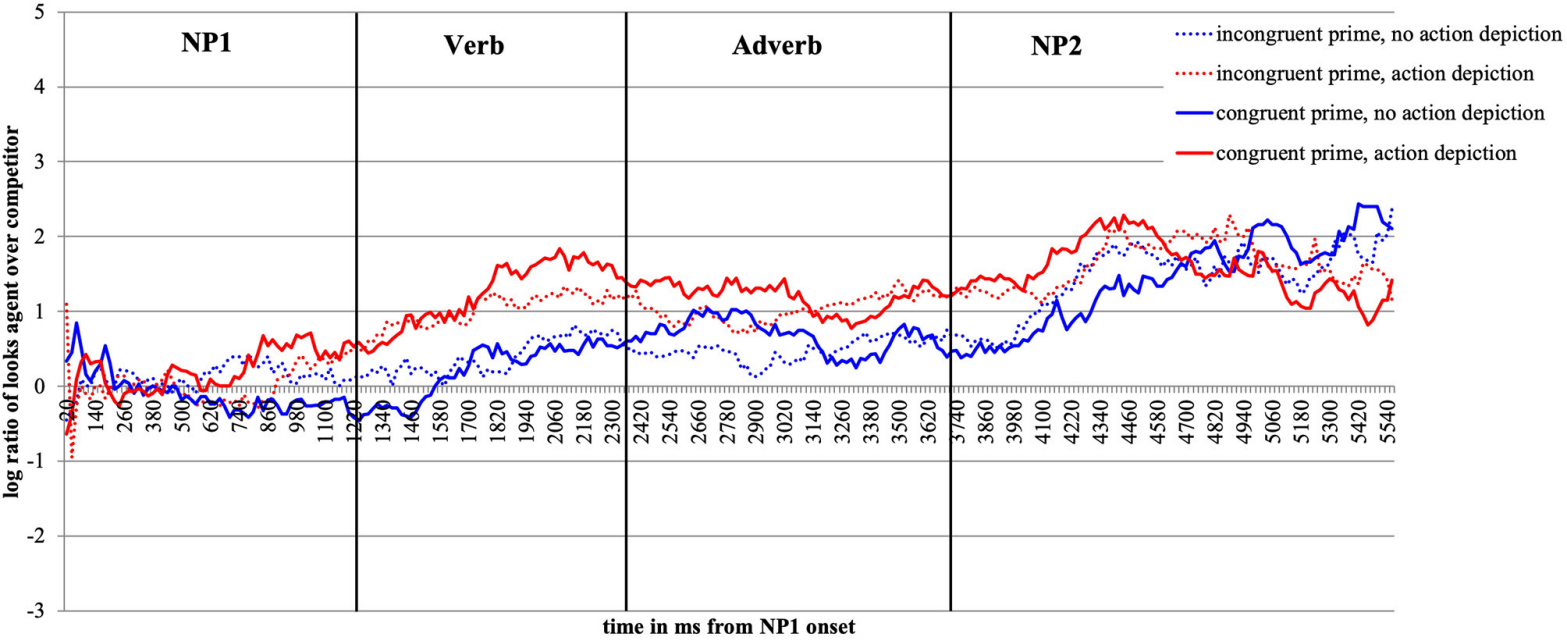
Time-course graph for Experiment 2 from NP1 onset until onset of the question display.

Figure 5 plots the time course of fixations to the target agent relative to the competitor character from the onset of NP1 until the end of the target display. The log ratios are plotted as a function of emotional prime and action depiction.

Figure 5 shows that upon hearing NP1, participants, as expected, look equally often at the agent and the competitor character. Upon encountering the verb, which makes available valence and action information, participants begin to look more at the agent than at the competitor as in Experiment 1. Moreover, under the conditions in which an action is depicted (red lines), the listeners' gaze clearly deviates from gaze in the no-action conditions (blue lines). This effect lasts from the verb until the target agent is mentioned (middle of the NP2).

When contrasting the incongruent (dotted lines) and congruent face prime conditions (solid lines), we note that they deviate. When no action is depicted, this happens from the end of the verb and into the adverb region (the solid is above the dotted blue line). When an action is present (red lines), the solid is above the dotted line from the middle of the verb onward until the middle of the adverb region. The presence of the emotionally congruent (vs. incongruent) prime thus draws more looks toward the target agent during the verb and the adverb region (recall that these are the critical word regions as they give away the action and the valence of the sentence). During the NP2 region, the red and the blue lines converge again as the agent is named and can thus be discriminated.

#### Inferential Eye-Movement Analyses

**Figure 6:**
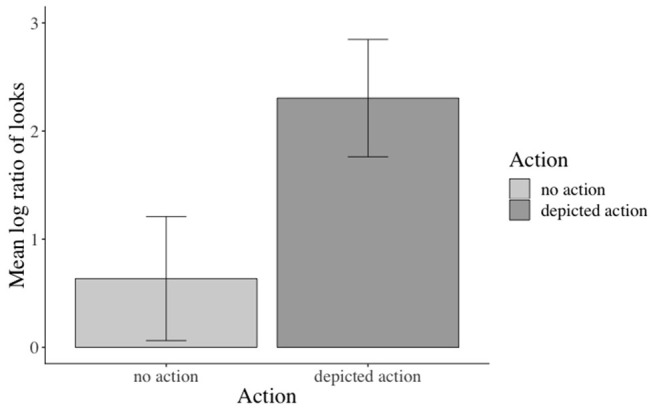
Significant main effect of action in the verb region. Error bars = 95% CIs.

**Figure 7:**
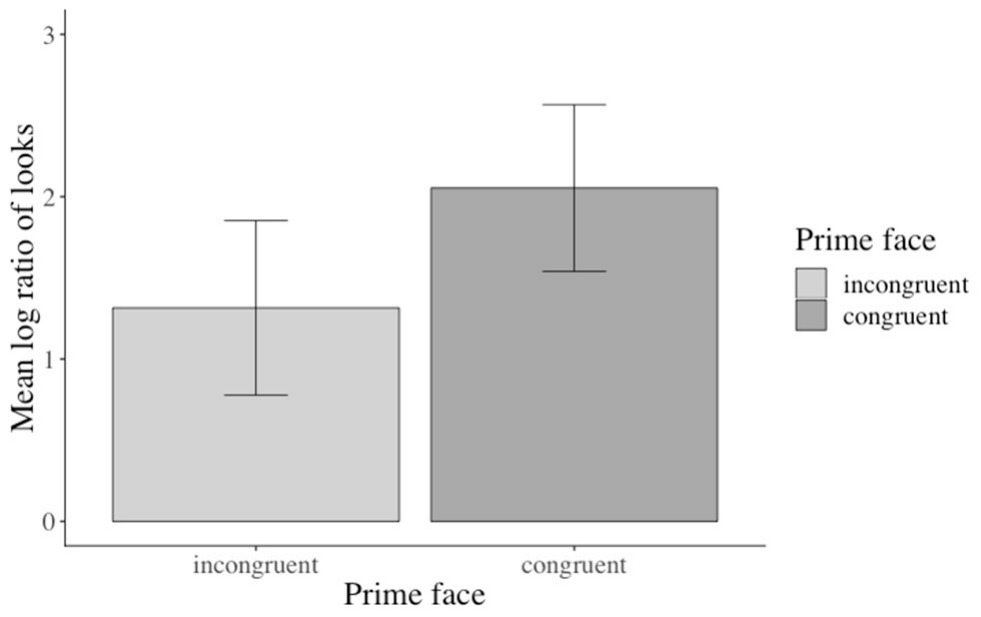
Significant main effect of emotional prime in the verb-adverb region. Error bars = 95% CIs.

Inferential statistics indicated a main effect of action: participants fixated the target agent significantly more than the competitor during all analyzed word regions when an action was (vs. was not) depicted, except for the NP1 region^11^ (i.e., verb region^12^: β: −0.826, SE: 0.1409, *df* : 577.300, *t*: −5.867, *p* < 0.01, see Figure 6). Moreover, a main effect of emotional prime emerged in the verb-adverb^13^ (β: −0.364, SE: 0.129, *df* : 522.3, *t*: −2.810, *p* < 0.01, see Figure 7) and the long region^14^ (β: −0.224, SE: 0.106, *df* : 572.7, *t*: −2.100, *p* < 0.05). Participants fixated the target agent significantly more than the competitor when the speaker's prime face was positive (vs. negative) upon hearing the verb-adverb and across the long region. The interaction between action and emotional prime was not significant (see the Supplementary Material for additional model parameters of all analyses).

#### Accuracy Results

Participants answered 96% of the comprehension question and 99.4% of the face recall question correctly. The analysis of the answers for the “who-does-what” comprehension questions^15^ yielded no significant effects of prime face or action.

### Discussion Experiment 2

The eye-tracking results reinforce the insight from Experiment 1—viz. that people use the depicted action toward discriminating the agent of the OVS sentence starting from the verb. In contrast to Experiment 1, we also observed a significant effect of the emotional prime in the verb-adverb and long region. As before, however, the emotional cue seems to be used to a lesser degree for sentence interpretation than the action depiction as the effects emerged only in the verb-adverb and long region and were not as pervasive as the action effect. Participants' accuracy in answering both the comprehension and face recall questions was high (no significant effects of the independent factors on accuracies were observed).

Experiments 1 and 2 replicated the effects of depicted actions on the assignment of thematic roles in non-canonical German OVS sentences. Participants reliably used depicted actions as contextual cues to anticipate the target agent, facilitating sentence processing in real time. Yet, whereas the schematic smiley did not affect real-time sentence processing, the natural emotional speaker's face led to a significant effect in the verb-adverb and the long region, i.e., across the whole sentence. Nevertheless, the emotional prime face still had a less pervasive effect than the depicted action. Our final study addressed this issue to examine the emotional prime effect in more detail. We will briefly address and motivate each change for Experiment 3 with regard to the results of Experiments 1 and 2. Note that Experiment 3 was conducted to maximize finding an effect of the emotional facial expression, resulting in more changes than for an incremental replication experiment.

**Figure 8:**
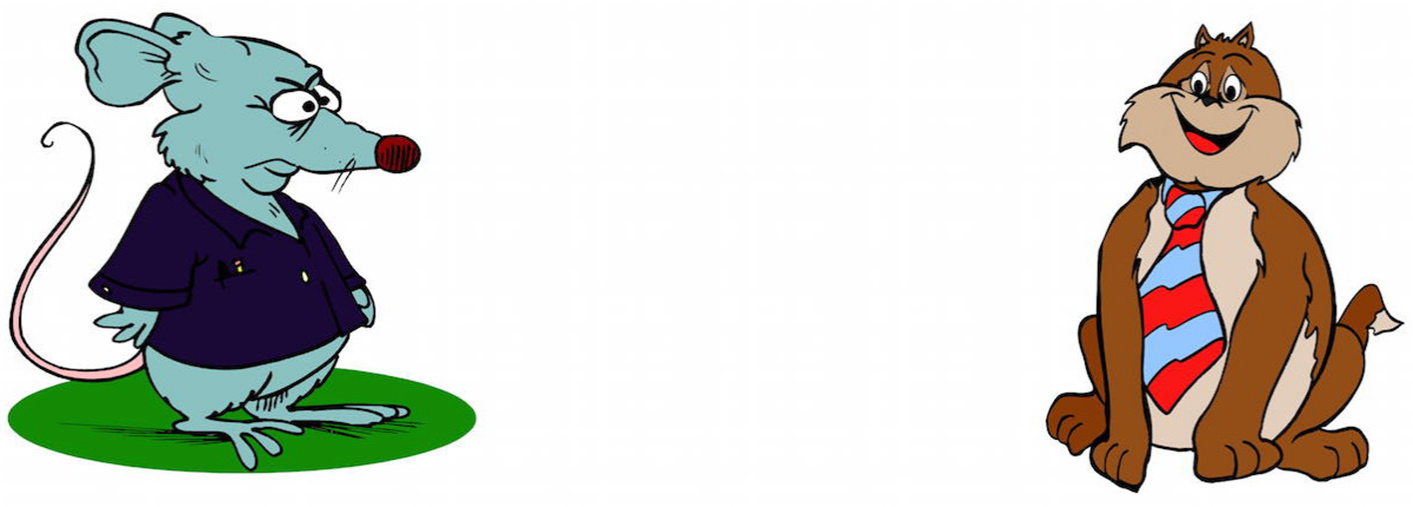
Example item from Experiment 3: emotional facial expressions of the competitor character (i.e., the rat) and the target agent (i.e., the cat) are increased in valence compared to items from Experiments 1 and 2 (cf., Figure 1).

Accordingly, we changed the design, materials, presentation, and task in Experiment 3 such that the focus was on the speaker's facial expression. We omitted the action factor and scenes depicted no actions. Additionally, we removed the middle character; target scenes only contained the happy-looking target agent and the grumpy-looking competitor character. Further, the characters' facial expressions were rendered more salient (see Figure 8). In line with this, the emotional facial prime was introduced in an explicit manner as the speaker of the sentences: Participants were told that it is a mother who be reading short sentences about the actions of cartoon characters to her child. With the prime face now being explicitly introduced as the speaker, we hoped to increase participants' emotional expectations about the following scene: A happy-looking speaker is more likely going to utter an emotionally congruent (vs. incongruent) event, which more likely features a happy (vs. grumpy) looking agent. The visual scene was reduced in complexity, giving participants the chance to focus on the linking of emotional valence between the visual and linguistic input. Moreover, this linking was made more explicit to participants, highlighting its ecological validity, by framing a real-world interactive setting between a mother and her child.

We hypothesized that the less pervasive effect of the emotional prime effect might be due to the different ways of cue presentation. While the depicted actions were present during real-time sentence processing, the emotional prime face was presented prior to encountering the target scene and sentence. In Experiment 3, we presented the speaker's facial expression as a prime because presenting it simultaneously together with the target scene while participants listen to the OVS sentence would have drawn attention away from the characters [unpublished pilot data from another study (2011) supports this view]. However, we decreased the scene preview time from 2,000 to 500 ms. This shortens the time for which the emotional prime must be retained in working memory, perhaps facilitating its effects on the processing of the target sentence.

A further change concerned participants' task. In Experiments 1 and 2, participants answered comprehension questions in the active or passive voice for who-is-doing-what-to-whom and/or questions about the emotional expression of the prime face and the feelings of the depicted characters. No time limit had been imposed and accuracy had been at ceiling. To assess whether participants kept the prime face in memory during the trials, in Experiment 3 we presented them with a screenshot of either the positive or the negative speaker face after *each* trial. Participants were instructed to verify *via* button press as quickly and accurately as possible whether the screenshot of the facial expression matched the speaker's expression in the prime video. We further assessed participants' reaction time for the assignment of thematic roles. On each trial, following the face verification, the target sentence was repeated in SVO structure. Participants were asked to verify as quickly and accurately as possible whether the OVS target sentence and the SVO sentence described the same event or not. A correct answer indicated participants had understood the thematic role relations in the OVS sentence. Finally, as we had 16 experimental items and relatively low power, we increased our sample from 40 to 64 participants.

With the implemented changes, the resulting simplified design and a stronger focus on the interactive and situated setting in the instructions, we expected to find a more pervasive effect of a speaker's emotional facial expression on the real-time processing and thematic role assignment of non-canonical OVS sentences, if, as Experiment 2 suggested, emotional facial expressions can indeed facilitate the assignment of thematic roles.

## Experiment 3

### Method and Design

#### Participants

A further 64 students of Humboldt-Universität zu Berlin, all native speakers of German with normal or corrected-to-normal vision, between 18 and 30 years (32 male, mean age: 23, SD age: 3.39) took part in the experiment. Participants were tested in the eye-tracking laboratory of Humboldt-Universität zu Berlin. One participant had to be excluded due to a technical problem. Participants received 8.50 Euro for their participation. All gave written informed consent. Ethics approval was given by the ethics board of the German association for linguistics (DGfS, Laboratory ethics approval, valid from September 17, 2016 to September 16, 2022).

#### Design

We are only reporting the changes compared to Experiment 2 here. We removed the depicted actions, and retained the factor emotional prime with two levels: congruent prime vs. incongruent prime. We additionally changed the face recall questions to prime face verification: participants are now asked to decide as quickly and accurately as possible if the speaker's prime face matches the posttrial picture of the speaker's face. Additionally, we changed the who-does-what question: participants heard a sentence either matching or mismatching the target OVS sentence in content, sentence structure, or in both. They decided as quickly and accurately as possible whether the content in the two sentences was identical or not. Participants answered with their left and right index fingers using yes/no buttons on a button box. The position of the yes/no buttons was counterbalanced across participants. One experimental session took about 50 min.

#### Materials

We removed the middle character (the patient) from the scenes, such that participants' attention is only divided between the target agent and the competitor character. Moreover, the target and competitor character's emotional facial expressions were improved such that the competitor character's face was very negative and the target agent's face very positive (see Figure 8).

For the prime face verification questions, a screenshot of the final frame of the speaker's emotional prime face video was taken and used in each condition: For all critical items in a list, the picture of the test face was positive in half of the trials and negative in the other half. Moreover, the test face matched the prime face in the video in half of the trials and mismatched in the other half of the trials. The filler trials were also followed by either a (mis) matching positive or (mis) matching negative emotional prime face picture. Across all trials, the valence and emotional match of the pictures used for prime face verification were counterbalanced within and across lists and participants.

We further created 16 SVO versions of the critical OVS item sentences for the sentence verification questions. All critical to-be-verified sentences matched in content, i.e., also in role relations and only mismatched in sentence structure, thus requiring a yes answer. Additionally, we created a verification sentence for each of the 24 filler sentences: The filler sentences either mismatched in sentence structure (*N* = 4), in content (*N* = 22), or matched in structure (*N* = 24) or in content (*N* = 6) with the to-be-verified sentence. Across all 44 critical item and filler trials, 24 of the verification sentences matched in structure and 20 mismatched. Also, half of the 44 verification sentences matched in content with the to-be-verified sentences and the other half mismatched in content. This resulted in half of the trials requiring a yes and half of them requiring a no answer for each participant. The same speaker (PK) that recorded the critical and filler sentences also recorded the sentences for the sentence verification question.

### Hypotheses

If we find more looks to the target agent (vs. the competitor) during the verb-adverb region in the positive congruent prime condition (vs. the negative incongruent prime condition), we could conclude that participants have (a) correctly assigned thematic roles even in the absence of depicted action cues and (b) have done so in a facilitative fashion with the help of the positive prime face. The reaction-time data should corroborate that participants are faster in verifying OVS/SVO event identity when the prime is congruent (vs. incongruent). Conclusions regarding the assignment of thematic roles are less clear if, however, we only find effects of the positive prime face in the eye tracking but not the reaction-time data. People would still have established an on-the-fly link between the positive face, the target agent's happy facial expression and the positive verb-adverb. But—in the absence of an emotional prime effect on the response times—we could not be sure that the emotional prime improved the post-trial comprehension of the role relations. By contrast, if we find an effect of the positive prime face in the response times but not in the eye-movement record, then we could conclude that the prime face helps participants to correctly assign thematic roles but that this effect takes time and participants cannot use it “on the fly” to ease the assignment of thematic roles.

### Procedure

**Figure 9:**
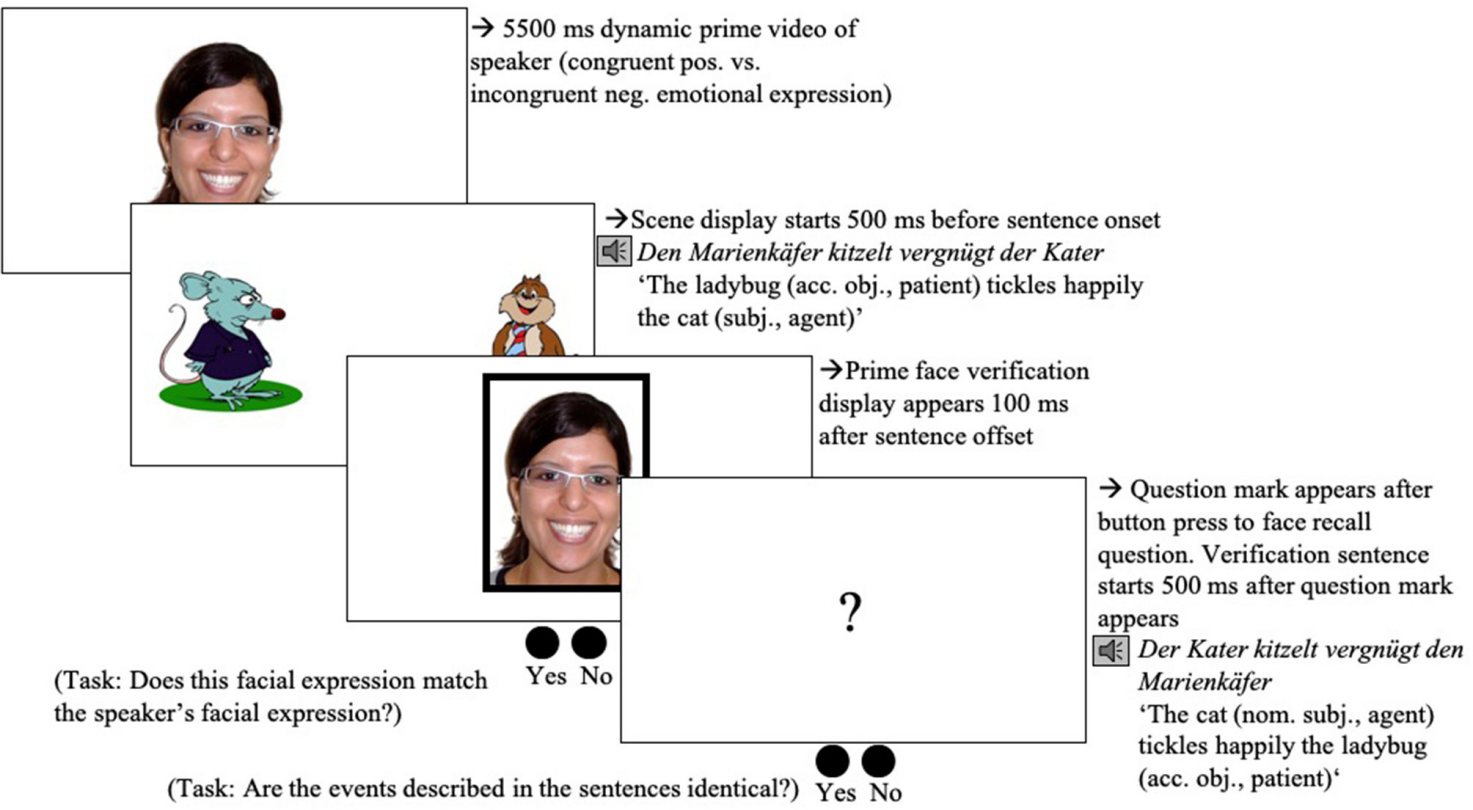
Procedure of a trial in Experiment 3. The example shows a “yes” response for the face-face verification, and for the OVS target-sentence/posttrial SVO sentence verification.

Each trial started with a fixation dot (not depicted in Figure 9), followed by the positive (vs. negative) emotional prime face video of the speaker of the following sentence. After the video (5,500 ms), participants encountered the scene showing the happy-looking target agent (the cat in Figure 8) and the grumpy-looking competitor (the rat in Figure 8) for 500 ms before the OVS sentence started to play. Participants looked at the scene while listening to the sentence. Following the sentence, the prime face verification display showed either a positive or a negative static frame from the prime face video. Participants indicated as quickly and accurately as they could if this facial expression matched the speaker's facial expression in the prime video. Following the button press, a question mark appeared on the screen. About 500 ms after the onset of this question mark, participants listened to the to-be-verified SVO sentence. They were asked to judge sentence identity as quickly and accurately as possible.

### Analyses

The analyses of the eye-tracking, accuracy, and response-time data followed the same procedure as for Experiments 1 and 2 (see Sections Eye Movements and Analyses) but the eye-tracking data analysis was performed on correctly answered trials only. The fixed factor for the eye-tracking analyses was emotional prime (congruent vs. incongruent). The fixed factors for the prime face verification accuracy and RT analysis were emotional prime (congruent vs. negative) and match (prime face—test face match vs. prime face—test face mismatch). The fixed factor for the sentence verification accuracy analyses was emotional prime (congruent vs. incongruent). The response-times for the prime face verification and the sentence verification questions (for the correctly answered trials only) were analyzed by mixed-effects models using log transformed reaction times as a dependent variable and prime and match (match was not a fixed factor in the sentence verification question because all critical items were followed by a matching SVO sentence) as fixed factors. Reaction times +/- 2.5 SD^*^ condition mean were treated as outliers and excluded from the analysis.

### Results Experiment 3

#### Descriptive Eye-Movement Results

**Figure 10:**
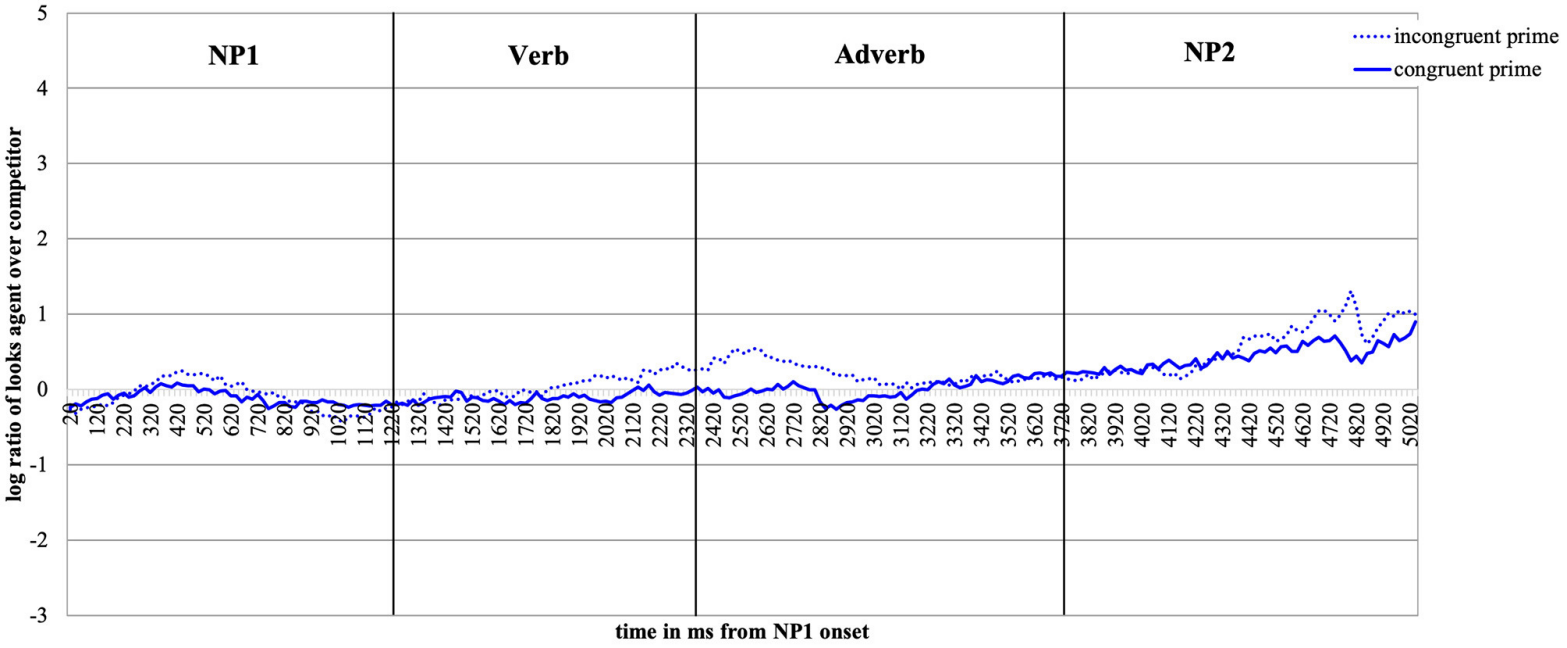
Time-course graph for Experiment 3 from NP1 onset until onset of the prime face verification display.

Figure 10 shows the time-course graph for Experiment 3. Whereas participants did not seem to have a preference to either look at the competitor or at the target agent during the first noun phrase and until the middle of the verb region, a preference to look more at the target agent emerged from the middle of the verb until the middle of the adverb region in the incongruent prime condition. Interestingly, this deviation between the solid and the dotted blue line shows that participants fixated the happy-looking target agent (vs. the grumpy-looking competitor) more after having been primed with a negative and sentence valence-incongruent (vs. positive valence-congruent) speaker face. At the end of the adverb region and until the end of the sentence, participants started to look at the target agent in both conditions when it was named in the NP2 region but descriptively more in the incongruent (vs. congruent) prime condition.

#### Inferential Eye-Movement Analyses

**Figure 11:**
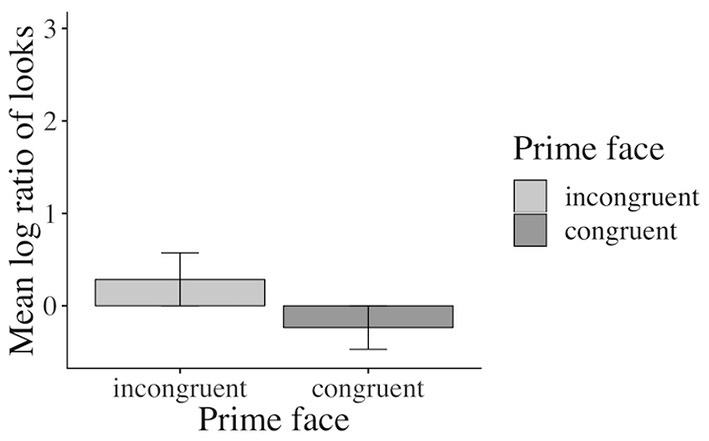
Significant main effect of prime face in the long region (correctly answered trials only, Experiment 3). Error bars = 95% CIs.

Accuracy in the prime face and sentence verification questions was lower (92 and 77% correct answers, respectively) than for the who-does-what questions (96% correct answers in both Experiments 1 and 2). Given the lower accuracies, we excluded incorrectly answered prime face verification and sentence verification trials from the analysis. The analyses revealed a significant main effect of emotional prime in the verb-adverb^16^ (β: 0.2749, SE: 0.1192, 16.0189, *t*: 2.307, *p* < 0.05) and in the long region^17^ (β: 0.25010, SE: 0.09261, *df* : 17.88568, *t*: 2.700 *p* < 0.05, see the Supplementary Material for additional model parameters of all analyses): participants in the verb-adverb region and across the sentence fixated the competitor character more after having seen a positive congruent (vs. negative incongruent) prime face and vice versa (see Figure 11)^18^.

#### Accuracy Results

**Figure 12:**
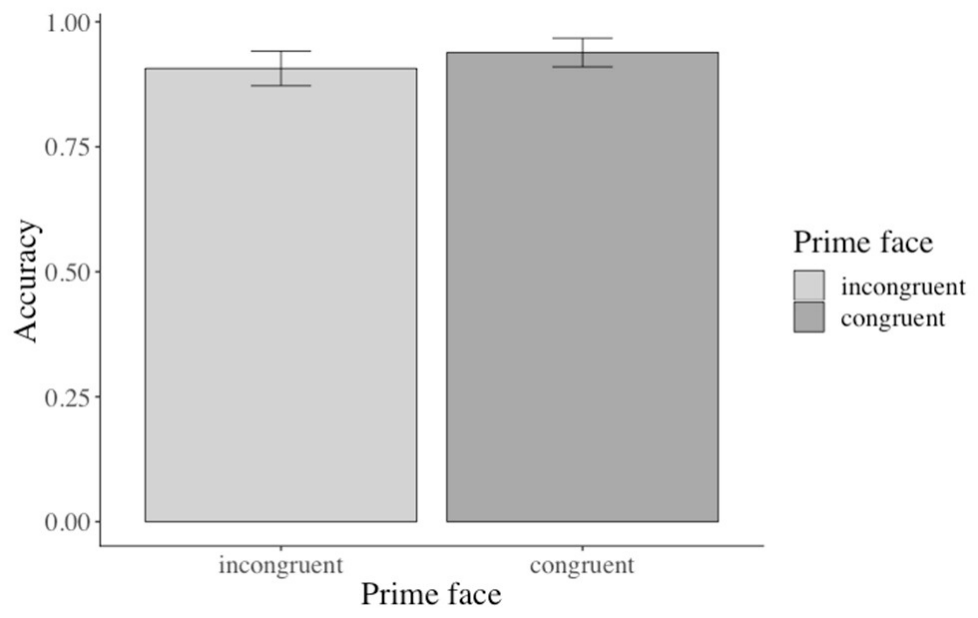
Marginal main effect of the prime face for the accuracy data in Experiment 3. Error bars = 95% CIs.

Across conditions, participants answered 92% of the prime face verification questions and 77% of the sentence verification questions correctly. The analysis revealed a marginal (β: −0.3842, SE: 0.2020, *z*: −1.902, *p* = 0.0571) main effect of prime face for the face verification questions^19^: participants answered more verification questions correctly when the prime face was congruent (47% vs. incongruent 43%, see Figure 12). Accuracies for match (vs. mismatch) conditions did not differ significantly. The accuracy analysis for the sentence verification question did not yield any significant effects of the manipulated factor^20^.

#### Reaction Time Results

**Figure 13:**
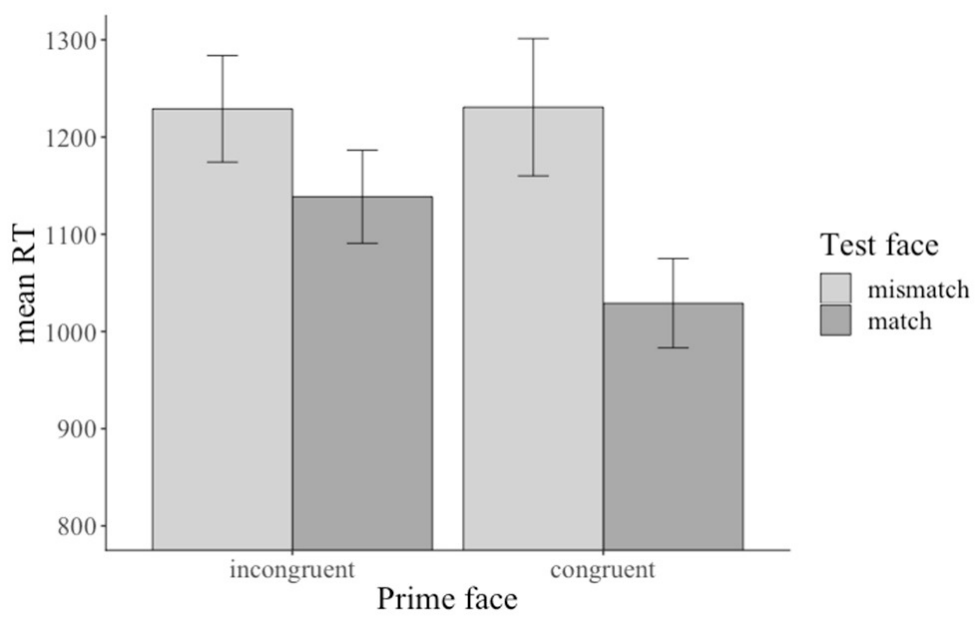
Significant main effects and interaction for the reaction time data in Experiment 3. The prime was congruent (vs. incongruent) with sentence valence. The test face either matched or mismatched with the prime face. Error bars = 95% CIs.

The reaction time analysis for the prime face verification question revealed a significant main effect of emotional prime (β: 0.030808, SE: 0.008537, *df* : 814.045317, *t*: 3.609, *p* < 0.001), a significant main effect of match (β: 0.058065, SE: 0.008648, *df* : 818.756839, *t*: 6.714, *p* < 0.001), and a significant emotional prime x match interaction (β: −0.028995, SE: 0.008649, *df:* 817.432855, *t*: −3.352, *p* < 0.001): participants responded faster when the prime face was congruent (vs. incongruent, see Figure 13) and when the prime face valence and the test face valence matched (vs. mismatched, see Figure 13) with each other. Moreover, they responded faster when the prime face was congruent and matched (vs. incongruent and mismatched) with the test face (see Figure 13)^21^. The reaction-time analysis for the sentence verification question did not reveal any significant effects of the manipulated factors.

### Discussion Experiment 3

In Experiment 3, we set the focus on the emotional prime face as a cue for language processing and highlighted the emotional valence and its relevance in the experimental materials, the task, presentation, and the design. We predicted that the increased focus on the emotional valence information would bring out the effect of the emotional prime face that we observed in Experiment 2 even more, i.e., participants were expected to anticipate the happy-looking target agent during the positively valenced verb-adverb and long region more when they had been primed with a congruent (happy vs. incongruent, sad) speaker facial expression. The emotional prime face did affect the looks to the characters in the scene, and this effect emerged in the same regions as in Experiment 2 (the verb-adverb and the long region). However, the directionality of the effect goes into the opposite-than-expected direction. Participants preferred to look at the grumpy-looking competitor character more after being primed with a sentence-congruent (positive) prime face and preferred to look at the happy target agent more after being primed with a sentence-incongruent (negative) prime face (Figures 10, 11). The reaction time and (to some extent) the accuracy of the face verification data, on the other hand, show that participants verified the positive test face picture faster and more accurately after being primed with a congruent (positive) speaker prime face (vs. incongruent negative). We discuss this finding in the general discussion (see Section The Reversed Priming Effect).

## General Discussion

In three visual-world eye-tracking experiments, we assessed whether participants' incremental comprehension of non-canonical OVS sentences is modulated to the same extent by referential cues (depicted actions) and non-referential cues, i.e., visual (social) cues (positive facial emotions that are arguably non-referentially linked to the sentence meaning).

### The Action Effect

In Experiments 1 and 2, participants made extensive use of the depicted action (vs. no action) for online sentence processing. They were more likely to anticipate the correct target agent (vs. a competitor) during OVS sentence processing in real time when the agent depicted in the scene was performing (vs. was not performing) the action mentioned in the sentence. We hence replicated existing findings demonstrating that a supportive visual context, which is referenced by the linguistic input, can rapidly facilitate OVS sentence processing and the assignment of thematic roles (cf., Knoeferle et al., 2005, 2008; Zhang and Knoeferle, 2012).

### The Positive Prime Effect

The positive prime face, by contrast, had only a significant effect on sentence comprehension when the prime face was human (Experiments 2 and 3), but not when it was a smiley (Experiment 1). Moreover, the effects of the emotional facial expression were less pervasive compared to the effects of the referenced depicted action.

The latter finding could be caused by the way the listeners link aspects of the scene to language. While the depicted action is mediated by its referring linguistic expression (the verb), matching the emotional valence of a preceding prime face to the target character's facial expression and to the valence of the adverb is arguably more complex. In our view, the greater complexity arises because, first of all, the listener must infer the valence of the emotional prime face, since no emotion labels are provided. Keeping the inferred emotion in memory, s/he then inspects the target scene and starts to interpret it in relation to the unfolding OVS sentence. Only when the valence of the emotional sentence becomes clear (during the verb and adverb regions), could the listener reactivate the previously seen emotional face and link the valence of the face to the emotional adverb and the happy smile of the target agent. In Experiment 2, this prime face-language connection enabled anticipatory looks to the target agent. We propose that effects of the non-referential cue arguably involve more processing steps than the effects of the referential cue on real-time assignment of thematic roles (the verb mediates the action and the associated agent that can fill a thematic role slot).

Alternatively, the effects of the emotional primes were less pervasive than of the actions because of differences in presentation. While the depicted action was present during sentence presentation, the emotional facial expression was shown and then removed before the onset of the target scene and sentence. Thus, whereas the listener had access to the depicted action throughout comprehension, the emotional prime face had to be held in working memory from the end of the prime presentation until the end of the trial. The effects of the emotional prime might be less pervasive because relating visual and linguistic input is arguably easier when both are presented together than when they are presented separately. This assumption is indirectly supported by Glenberg and Robertson's (1999) indexical hypothesis. It assumes that the referencing (or indexing) of language to the visual context is easier when both are co-present compared to when they are presented separately. That said, other studies have reported emotional face effects in a serial prime-target paradigm. The prime and target presentation in Carminati and Knoeferle (2013) were—just like in our studies—serial in nature.

Another reason for why the effects of the non-referential cue were less pervasive could be the different sentence structures investigated. Recall that Carminati and Knoeferle (2013) and Münster et al. (2014) investigated emotional priming effects on the processing of SVO sentences. Processing structurally challenging non-canonical OVS sentences and assigning thematic roles, as was the case in our studies, is arguably a cognitively more demanding task than reconciling a prime face with the semantic meaning and valence of a canonical SVO sentence. Linking a speaker's emotional facial expression to a corresponding emotionally valenced adverb and a character's emotional facial expression while assigning thematic roles in a non-canonical OVS sentence might account for the subtler effects compared to the study by Carminati and Knoeferle (2013) and Münster et al. (2014).

However, while being less pervasive than the effects of the depicted action in Experiment 2, the emotional prime effects provide some support for the view that the valence of a prime face can also facilitate the real-time assignment of thematic roles in non-canonical German OVS sentences.

### The Naturalness of the Emotional Prime Face

The analyses also revealed significant effects of the dynamic *natural* (but not the smiley) emotional facial expressions. One possible reason for that could lie in the changes in emotional prime presentation between Experiments 1 and 2. While the happy utterance-congruent smiley (Experiment 1) contrasted with a (nonemotional utterance-unrelated) static red star, the valence congruence manipulation in Experiment 2 was achieved by contrasting a woman's happy (utterance-congruent) and sad (incongruent) facial expressions. Hence, we contrasted an emotional with a non-emotional prime in Experiment 1 but one woman's facial expression of contrasting valences in Experiment 2. In order to strengthen our claim that only the naturalness of the emotional prime face elicited an effect in Experiment 2 but not 1, the happy smiley prime in Experiment 1 would have to be contrasted directly with a sad smiley prime instead of a red star in a follow-up study. Additionally, using valence-contrasting faces in Experiment 2 may have increased the overall awareness of emotions in the study and boosted prime effects compared to Experiment 1. This might additionally be the case because in Experiment 2, the focus on emotions was further increased by occasionally asking participants to comment on the characters' feelings and to recall the valence of the prime face.

However, it could also be argued that the longer exposure duration of the natural facial prime (5,500 vs. 1,750 ms for the schematic smiley) caused its better integration into sentence processing. A longer exposure to the emotional expression might have led to more in-depth processing of the emotional content although the studies that have varied stimulus duration (from as short as 50 ms to 10 s) of emotional facial expressions found no effect of the duration manipulation on emotion perception and recognition for happy faces (Calvo and Lundqvist, 2008; Codispoti et al., 2009). Hence, the stronger effects of the natural dynamic facial expression are likely due to its higher ecological validity and not the longer stimulus exposure.

Ecological validity might have contributed to a better integration of the human (compared to the smiley) prime face into the real-time processing of the OVS sentence. The target sentence was spoken by a female voice, and the gender match with the face prime might have encouraged participants to more readily perceive the happy-looking woman as the speaker than a smiley. That this is likely the case is also supported by “Theory of Mind” assumptions (e.g., Premack and Woodruff, 1978). Using the facial expressions of our interlocutors, we attribute our own mental states to others and expect the other person to act like we would have acted with a similar facial expression. Additionally, the use of more naturalistic real-world stimuli in experiments, especially those on emotion and language, is recommended (e.g., Adolphs, 2006). Many studies on human (social) cognition often use morphed, synthetic, or static faces. The social link that two humans establish during communication may not emerge in the same way when one partner is not human (e.g., a synthetic face or a smiley). This conclusion is also supported by recent behavioral and ERP evidence showing that schematic/cartoon faces are not processed as deeply as a natural facial expression and that more attentional resources are allocated to natural faces (e.g., Schindler et al., 2017; Kendall, 2019; Zhao et al., 2019; see Section Non-Referential (Visual) Social Cues: Facial Emotions). In our Experiment 2, the natural facial prime may have set up the expectation of a human speaker more than the smiley in Experiment 1, eliciting a better integration with the target agent's facial expression and the positive sentence.

### The Reversed Priming Effect

However, when the emotional facial expressions of the competitor character and target agent were more salient, the anticipatory effect of the congruent prime face reversed. Participants were more likely to fixate the grumpy-looking competitor character (vs. happy-looking target agent) during the verb-adverb and long region when having been primed with a positive (vs. negative) speaker face. We did not predict this reversal but identified possible reasons *post-hoc*.

To recap, in Experiment 2, the visual scene that participants saw while listening to the positively valenced sentence contained the happy-looking target agent alongside a neutral-looking patient and a slightly grumpy-looking competitor character. In Experiment 3, we omitted the neutral-looking patient and increased the valence of both the target agent's and the competitor character's emotional facial expressions. This means that the slightly grumpy-looking competitor character was given a really grumpy face (e.g., by lowering the corners of the mouth and lowering and tilting the eyebrows) while the happy-looking target agent's face was made even happier looking (e.g., increasing the size of the eyes, opening the smile, and raising the eyebrows). That is, in Experiment 3, the emotional valence of the target scene was foregrounded and the valence highlighted.

We assume that in both experiments, the congruent happy speaker prime face raised expectations toward an upcoming happy event. In Experiment 2, following this congruent prime face, and with the expectations toward a positive event in mind, participants faced a scene in which the emotionally most salient character is the happy-looking target agent. The slightly grumpy-looking character might not have been as emotionally salient and might hence not have violated the primed positive expectations. Hearing the verb and adverb describing the happy event in the scene, participants quickly used their expectations and the congruent positively valenced linguistic input to direct their fixations toward the also happy-looking target agent. When that agent was named, their expectation that this is indeed the character carrying out the positive action is confirmed.

In contrast, in Experiment 3, following the congruent prime face, participants encounter a scene featuring a very grumpy-looking and a very happy-looking character. Both characters' facial expressions might be salient, but it could be that the really grumpy-looking character drew more attention than the really happy-looking character. This idea is supported by evidence suggesting that negative emotional facial expressions and events draw more attention and are better memorized than positive faces and events [at least for younger adults (e.g., Grühn and Scheibe, 2008; Lamy et al., 2008; Finn et al., 2012; Bach et al., 2014)].

The very grumpy-looking character might also violate the positive expectations more than it did in Experiment 2 (in which the face was only slightly grumpy). Rothermund et al. (2011), for example, showed incongruency effects in affective processing in emotionally mismatching situations. Further findings show that attention is directed to materials opposite in emotional valence to the perceiver's current focus of attention (Rothermund, 2003; Rothermund et al., 2008). This attentional state moreover can but does not have to be explicitly induced by, for instance, imagining personal positive or negative events or watching emotionally valenced movies (Schwager and Rothermund, 2013). In Schwager and Rothermund (2013), participants first imagined a personal emotional situation (Experiment 1) or watched positive or negative emotional movie clips (Experiment 2). Following the imagination or movie, they performed a visual search task (Experiment 1) or an emotional Stroop task (Experiment 2) featuring positive and negative target words (Experiment 1) or pictures (Experiment 2). The results showed that participants were more accurate in both experiments for opposite-valence targets. Moreover, in both experiments, the reaction times suggested that participants were also faster in detecting opposite-valence targets.

A similar interference effect has recently been reported for real-time language processing (Guerra and Knoeferle, 2017, 2018). In an eye-tracking reading study, Guerra and Knoeferle (2017) investigated how visually perceived spatial distance influences the interpretation of social relationships between agents and patients in sentence processing. Visually perceived spatial distance was established *via* pairs of playing cards, which either moved closer together or farther apart. The nouns referring to the agent and patient in the following German target sentence were printed each on one of the two cards. The card movements primed the written target sentence either expressing a friendly (i.e., close) or an unfriendly (i.e., far) relationship between the agent and patient, such as *Sandra met her aunt cheerfully/grumpily at the health center* (translation). In Experiment 1, in these sentences, the adverb expressing the social relation between the protagonists (i.e., *Sandra* and *her aunt*) appeared after the second noun (i.e., *aunt*). The results revealed shorter reading times in the adverb region when the cards moved close together and the sentence expressed a friendly relationship between protagonists compared to when prime card movement and target sentence did not match in social meaning. Hence, participants experienced a facilitation effect in sentence reading. However, in Experiment 2, Guerra and Knoeferle (2017) decreased the temporal distance between the card presentation and the (mis)matching adverb by moving the adverb in the sentence from after to in front of the second noun phrase (i.e., *Sandra met cheerfully/grumpily her aunt)*. The results revealed longer reading times for matching vs. mismatching social meaning. Thus, participants experienced an interference effect. The authors argue that the shift from facilitation to interference is due to an increased competition between two strongly activated representations of the same conceptual representation: When the previously seen playing cards prime the activation of the concept of un/friendliness and the adverb is encountered late after the agent–patient relationship has been established, sentence processing is facilitated. However, when the adverb is encountered before the second noun phrase, the concept of un/friendliness is activated earlier and competes in activation with the same concept activated by the matching playing cards. In the latter case, sentence processing took longer.

This reversal might be similar to the reversed priming effect in the present Experiments 2 and 3: whereas we see increased fixations to the happy-looking target agent in the adverb region in Experiment 2, we see increased fixations to the grumpy-looking competitor agent in the adverb region in Experiment 3. In Experiment 3, in contrast to Experiment 2, we not only increased the salience of the target agent and the competitor agent's opposing emotionally valenced facial expressions, but also we decreased the preview time of the target scene from 2,000 to 500 ms. Just like in Guerra and Knoeferle (2017), two conceptual representations might be competing for activation because they were activated in temporal proximity, thus reversing the effect. Crucially, in their final experiment, when Guerra and Knoeferle (2017) used the German sentences from their first experiment and inserted an additional neutral word between the second noun and the adverb (transl.: e.g., *Sandra met her aunt unequivocally cheerfully at the health center)*, they again found facilitation effects. Hence, the activation time between two similar conceptual representations seems to be crucial for the directionality of the effects regarding real-time language comprehension.

Taken together, the reversal of the result pattern that we see in Experiment 3 could (a) be due to the competitor character's highlighted negative facial expression, which violated the primed positive expectations and the positively valenced sentence. This explanation is also in line with studies showing that semantically inconsistent objects in scenes attract more attention (i.e., more fixations) than consistent objects based on the viewer's expectations and world knowledge about what a particular scene usually contains [e.g., a cocktail glass vs. a microscope in a kitchen scene (Henderson et al., 1999)]. However, following Guerra and Knoeferle (2017, see also 2018), the reversal of the result pattern that we see in Experiment 3 could (b) be also due to the timing of stimulus presentation in our experiments. Since we implemented both changes (i.e., the increase in emotional salience and the reduced target scene preview time), further investigations need to tease apart which of the two factors is responsible for the reversal of the effect in Experiment 3 compared to Experiment 2.

### Real-Time Assignment of Thematic Roles *via* Emotional Facial Expressions

The results from Experiment 2 suggest that participants use the speaker's emotional facial expression to anticipate the correct target agent when they can link the facial expression to the verb and adverb of the sentence, thus filling the agent role slot in real-time processing before the target agent is mentioned. Accuracy scores in Experiment 2 were at ceiling. These ceiling effects might have overshadowed potential off-line effects of the emotional prime face.

Experiment 3, however, seems to paint a slightly different picture compared to our initial interpretation of the results. In Experiment 3, in order to verify whether the content of the SVO and OVS sentences is the same, participants need to have correctly assigned the thematic roles in the sentence. Although this task was more difficult for participants than the passive who-does-what questions from Experiment 2 (77 vs. 96% correct answers), no effects of the prime face on sentence verification accuracy emerged. Experiment 3's null effect in the sentence verification accuracy plus the reversed result pattern in the eye-tracking data calls our interpretation that the positive prime face facilitates real-time assignment of thematic roles (Experiment 2) into question.

What the eye-tracking data does tell us though is that the linguistic input together with the emotional facial expression modulates attention to the characters in the scene, since the first effects emerged after the NP1 region, triggered by the verb and the adverb. Whether these effects are, however, specific to the assignment of thematic roles is less clear. The reversal of the result pattern in Experiment 3 could simply be due to the enhanced negative facial expression of the competitor character in the scene (see Section The Reversed Priming Effect). It could mean that the assignment of thematic roles can be facilitated by using the expectations based on the face in real time, if the visual context does not portray strongly opposing valence information (i.e., an only slightly grumpy-looking competitor, a neutral patient, and a happy-looking target agent) and/or if similar conceptual representations do not compete for activation in temporal proximity. The expectations to what should come next might be violated by seeing a valence-incongruent character on the screen next to a valence-congruent character. That contrast might have drawn listeners' attention to the valence mismatching (vs. matching) character when the positive verb-adverb region confirmed the expectations derived from the positive prime face (cf., Henderson et al., 1999; Schwager and Rothermund, 2013).

Alternatively, the effects we find in Experiment 2 are due to linking the positive facial expression to the positive sentence, which in turn directs attention to the character with the valence-matching facial expression without any assignment of thematic roles involved. Since the off-line effects did not support the real-time emotional prime effects, it might be argued that participants did not assign thematic roles based on the match between the emotional prime face, the positive sentence, and the target agent's facial expression, but merely performed a “valence match” between the different positively valanced information types. However, note that this conclusion cannot be drawn for the depicted action effect. Even though our data does not reveal significant off-line effects, previous research indicates that depicted actions can clearly be used to facilitate the assignment of thematic roles in real time and posttrial (e.g., Knoeferle et al., 2005, 2008). Our results suggest that utterance-congruent emotional facial expressions of a speaker can modulate attention during real-time language processing. Further research needs to investigate whether emotional face primes also facilitate the assignment of thematic roles.

Finally, the diverging results between Experiments 2 and 3 have crucial implications for psycholinguistic visual-world research: Even slight changes in the materials and timing could change the pattern of results in unexpected ways (e.g., Guerra and Knoeferle, 2014; Knoeferle et al., 2014). Future research may want to replicate these timing- and stimulus-related changes in the directionality of the observed effects and further assess timing and stimulus variability.

## Conclusions

Our results provide some support for the view that a natural facial expression of emotions elicited a priming effect on the interpretation of emotionally valenced OVS sentences (in contrast to a schematic smiley). Crucially, our studies show that different kinds of information in a visual context and distinct language-world relations yield distinct visual context effects on language processing. As one reviewer pointed out, it is, of course, also possible to establish different degrees of referentiality by using different linguistic cues, i.e., the emotion verb *like* is arguably non-referential in contrast to the action verb *kick*. Even though this would be an interesting issue to explore and likewise foster our insights on different degrees of referentiality, limiting referentiality vs. non-referentiality to linguistic expressions alone does not allow us to pit different extralinguistic (social) cues, i.e., emotional facial expressions and depicted actions, against each other. Interestingly, Experiment 3 showed that the exact nature of the situation in which emotional facial cues are embedded seems to influence the directionality of the effects. Clearly, more research is needed, which explores the effects of referential and non-referential (e.g., social) cues and their relation to each other in real-time language processing. Our studies represent a first step in going beyond the integration of referential cues in language comprehension and suggest that subtler and crucially also social cues, such as the emotional facial expression of a speaker, can affect real-time language comprehension. The reported findings pave the way for extant real-time language processing accounts to include the effects of non-referential (social) visual cues, their modulation by the naturalness of facial expressions, and their relative effect differences compared with referential (action) cues.

## Data Availability Statement

The raw data supporting the conclusions of this article can be made available upon request to the first author.

## Ethics Statement

The studies involving human participants were reviewed and approved by University of Bielefeld ethics board (Vote 2013-007) and ethics board of the German association for linguistics (DGfS, valid from 17.09.2016 to 16.09.2022). The patients/participants provided their written informed consent to participate in this study. Written informed consent was obtained from the individual(s) for the publication of any potentially identifiable images or data included in this article.

## Author Contributions

PK and KM designed the experiments and wrote the manuscript. KM conducted the studies and analyzed the data. The data are available upon request. Analyses of the data from Experiments 1 and 2 have previously been published in Münster (2016) and in parts in Münster et al. (2015). All authors contributed to the article and approved the submitted version.

## Conflict of Interest

The authors declare that the research was conducted in the absence of any commercial or financial relationships that could be construed as a potential conflict of interest.

## Publisher's Note

All claims expressed in this article are solely those of the authors and do not necessarily represent those of their affiliated organizations, or those of the publisher, the editors and the reviewers. Any product that may be evaluated in this article, or claim that may be made by its manufacturer, is not guaranteed or endorsed by the publisher.

## Footnotes

^1^Note that although reaction times would have been informative, we did not use this measure in Experiments 1 and 2 due to design consistency and comparability with a child language study.

^2^Recall that these were the critical word regions as the verb relates to the action and the adverb reveals the emotional valence of the sentence.

^3^Best-fitting model: log_ratio ~ prime ^*^ action + (1 | participant) + (1 | item).

^4^Best-fitting model: log_ratio ~ prime ^*^ action + (1 + action | participant) + (1 + action | item).

^5^Best-fitting model: log_ratio ~ prime^*^ action + (1 + action | participant) + (1 + action | item).

^6^Best-fitting model: log_ratio ~ prime ^*^ action + (1 | participant) + (1 | item).

^7^Best-fitting model: log_ratio ~ prime^*^action + (1+action | participant) + (1+action+prime:action| item).

^8^Best-fitting model: log_ratio ~ prime ^*^ action + (1 | participant) + (1 | item).

^9^Best-fitting model: answer ~ prime^*^ action ^*^ voice + (1 | participant) + (1 | item).

^10^The woman portraying the facial expressions gave informed consent regarding the use and publication of her facial expressions for research purposes related to our studies.

^11^Best-fitting model: log_ratio ~ prime ^*^ action + (1 + action | participant) + (1 | item).

^12^Best-fitting model: log_ratio ~ prime ^*^ action + (1 + prime | participant) + (1 + prime | item).

^13^Best-fitting model: log_ratio ~ prime ^*^ action + (1 + action | participant) + (1 + action | item).

^14^Best-fitting model: log_ratio ~ prime ^*^ action + (1 | participant) + (1 | item).

^15^Best-fitting model: answer ~ prime ^*^ action + (1 + action | participant) + (1 | item).

^16^Best-fitting model: log_ratio ~ prime + (1 + prime | participant) + (1 + prime | item).

^17^Best-fitting model: log_ratio ~ prime + (1 + prime | participant) + (1 + prime | item).

^18^Including all trials (correctly and incorrectly answered) in the analysis yielded a significant main effect in the long region (*p* <0.001), the effect in the verb-adverb region did not reach significance, but the result pattern stays identical.

^19^Best-fitting model: answer ~ prime + (1 + prime | participant) + (1 | item).

^20^Best-fitting model: answer ~ prime + (1 + prime | participant) + (1 | item).

^21^Best-fitting model: log_RT ~ prime ^*^ match + (1 | participant) + (1 | item).
