# Supplementary material for: Integration of Social Context vs. Linguistic Reference During Situated Language Processing
Source: Front Psychol. 2021 Aug 2;12:547360. doi: 10.3389/fpsyg.2021.547360 (PMC8365155; doi:10.3389/fpsyg.2021.547360)
Supplement: Supplementary file 1 [file Data_Sheet_1.pdf]

Supplementary Material 1

Fixed effect model parameters for the best fitting models (run with lme4 version 1.1-21).

Section 2.5.2 (Eye-tracking - Experiment 1)

NP1 region: log_ratio ~ prime * action + (1 | participant) + (1 | item)

	ß	SE	df	t	*p*
Intercept	0.02824	0.29805	18.73849	0.095	0.926
Prime	0.05254	0.15780	581.99999	0.333	0.739
Action	-0.17416	0.15780	581.99999	-1.104	0.270
Prime * Action	0.04636	0.15780	581.99999	0.294	0.769

Verb region: log_ratio ~ prime * action + (1 | participant) + (1 | item)

	ß	SE	df	t	*p*
Intercept	1.50802	0.27536	17.56726	5.477	<0.0001
Prime	-0.01062	0.14140	582.00000	-0.075	0.940
Action	-0.97390	0.14140	582.00000	-6.888	<0.0001
Prime * Action	-0.01219	0.14140	582.00000	-0.086	0.931

Adverb region: log_ratio ~ prime * action + (1 + action| participant) + (1 + action | item)

	ß	SE	df	t	*p*
Intercept	2.3641	0.2619	19.6622	9.026	<0.0001
Prime	-0.1305	0.1406	534.2303	-0.928	0.35362
Action	-1.3258	0.2977	16.3705	-4.453	<0.001
Prime * Action	0.1772	0.1406	534.2303	1.260	0.20826

Verb-Adverb region: log_ratio ~ prime * action + (1 + action| participant) + (1 + action | item)

	ß	SE	df	t	*p*
Intercept	1.86617	0.26314	19.00270	7.092	<0.0001
Prime	-0.11243	0.12518	531.49224	-0.898	0.369516
Action	-1.13030	0.23806	18.21638	-4.748	<0.001
Prime * Action	0.08387	0.12518	531.49224	0.670	0.503138

NP2 region: log_ratio ~ prime * action + (1 | participant) + (1 | item)

	ß	SE	df	t	*p*
Intercept	3.53083	0.21502	18.74146	16.421	<0.0001
Prime	0.03708	0.12024	582.00000	0.308	0.758
Action	-0.53953	0.12024	582.00000	-4.487	<0.0001
Prime * Action	0.09036	0.12024	582.00000	0.752	0.453

Long region: log_ratio ~ prime * action + (1 + action | participant) + (1 + action + prime:action | item)

	ß	SE	df	t	*p*
Intercept	1.73120	0.20140	20.57393	8.596	<0.0001
Prime	-0.01002	0.09251	106.75303	-0.108	0.913949
Action	-0.65575	0.14390	15.91688	-4.557	<0.001
Prime * Action	0.07596	0.09574	24.90352	0.793	0.435067

Section 2.5.3 (Accuracy - Experiment 1)

Answer ~ prime* action + (1 | participant) + (1 | item)

	ß	SE	z-value	*p*
Intercept	3.9362	0.4608	8.541	<0.0001
Prime	-0.2438	0.2261	-1.079	0.281
Action	-0.3067	0.2250	-1.363	0.173
Prime * Action	0.2245	0.2252	0.997	0.319

Answer ~ prime* action * voice + (1 | participant) + (1 | item)

	ß	SE	z-value	*p*
Intercept	5.7926	25.6868	0.226	0.822
Prime	1.1291	25.6817	0.044	0.965
Action	-1.5208	25.6816	-0.059	0.953
Voice	2.0910	25.6818	0.081	0.935
Prime * Action	-1.1611	25.6817	-0.045	0.964
Prime * Voice	1.7141	25.6817	0.067	0.947
Action * Voice	-0.9553	25.6817	-0.037	0.970
Prime * Action * Voice	-1.7233	25.6817	-0.067	0.946

Section 3.4.2 (Eye-tracking – Experiment 2)

NP1 region: log_ratio ~ prime* action + (1 + action | participant) + (1 | item)

	ß	SE	df	t	*p*
Intercept	0.23687	0.19933	14.10000	1.188	0.254
Prime	-0.09007	0.16081	536.90000	-0.560	0.576
Action	-0.15208	0.16502	39.00000	-0.922	0.362
Prime * Action	0.23179	0.16081	537.00000	1.441	0.150

Verb region: log_ratio ~ prime * action + (1 + prime| participant) + (1 + prime| item)

	ß	SE	df	t	*p*
Intercept	1.4689	0.2578	19.6000	5.698	<0.0001
Prime	-0.1499	0.2027	18.8000	-0.740	0.469
Action	-0.8267	0.1409	577.3000	-5.867	<0.0001
Prime * Action	0.1739	0.1409	577.2000	1.234	0.218

Adverb region: log_ratio ~ prime* action + (1 + action | participant) + (1 | item)

	ß	SE	df	t	*p*
Intercept	1.61420	0.26532	18.20000	6.084	<0.0001
Prime	-0.26884	0.14800	575.70000	-1.816	0.069817
Action	-0.67348	0.18523	43.60000	-3.636	<0.001
Prime * Action	-0.02159	0.14801	575.80000	-0.146	0.884067

Verb-Adverb region: log_ratio ~ prime* action + (1 + action | participant) + (1 + action| item)

	ß	SE	df	t	*p*
Intercept	1.68671	0.23407	21.10000	7.206	<0.0001
Prime	-0.36427	0.12963	522.30000	-2.810	<0.01
Action	-0.74186	0.17723	18.80000	-4.186	<0.001
Prime * Action	0.05572	0.12963	522.20000	0.430	0.667503

NP2 region: log_ratio ~ prime* action + (1 | participant) + (1 | item)

	ß	SE	df	t	*p*
Intercept	2.43306	0.15369	15.20000	15.831	<0.0001
Prime	-0.01047	0.12974	574.30000	-0.081	0.93573
Action	-0.50233	0.12974	574.60000	-3.872	<0.001
Prime * Action	0.13527	0.12974	574.40000	1.043	0.29756

Long region: log_ratio ~ prime* action + (1 | participant) + (1 | item)

	ß	SE	df	t	*p*
Intercept	1.43814	0.13883	15.90000	10.359	<0.0001
Prime	-0.22428	0.10682	572.70000	-2.100	<0.05
Action	-0.41207	0.10682	573.10000	-3.858	<0.001
Prime * Action	0.08385	0.10682	572.80000	0.785	0.432778

Section 3.4.3 (Accuracy – Experiment 2)

Answer ~ prime* action + (1 | participant) + (1 | item)

	ß	SE	z-value	*p*
Intercept	3.82346	0.49288	7.757	<0.0001
Prime	-0.31218	0.22553	-1.384	0.166
Action	-0.47471	0.48876	-0.971	0.331
Prime * Action	-0.04383	0.22546	-0.194	0.846

Section 4.5.2 (Eye-tracking – Experiment 3)

NP1 region: log_ratio ~ prime + (1 + prime | participant) + (1 | item)

	ß	SE	df	t	*p*
Intercept	-0.2324	0.1568	16.1751	-1.482	0.157
Prime	0.2229	0.1413	15.1169	1.578	0.135

Verb region: log_ratio ~ prime + (1 + prime | participant) + (1 | item)

	ß	SE	df	t	*p*
Intercept	-0.08732	0.17657	14.66108	-0.495	0.628
Prime	0.19417	0.13591	624.93007	1.429	0.154

Adverb region: log_ratio ~ prime + (1 | participant) + (1 | item)

	ß	SE	df	t	*p*
Intercept	0.03515	0.16706	15.08514	0.210	0.836
Prime	0.23302	0.14754	626.72845	1.579	0.115

Verb-Adverb region: log_ratio ~ prime + (1 + prime | participant) + (1 + prime | item)

	ß	SE	df	t	*p*
Intercept	-0.1135	0.1330	15.3112	-0.853	0.4068
Prime	0.2749	0.1192	16.0189	2.307	<0.05

NP2 region: log_ratio ~ prime + (1 | participant) + (1 | item)

	ß	SE	df	t	*p*
Intercept	0.96951	0.17930	54.85442	5.407	<0.0001
Prime	0.05537	0.14113	595.70571	0.392	0.695

Long region: log_ratio ~ prime + (1 + prime| participant) + (1 + prime | item)

	ß	SE	df	t	*p*
Intercept	0.01278	0.09453	16.43765	0.135	0.8941
Prime	0.25010	0.09261	17.88568	2.700	<0.05

Including incorrect trials: Verb-Adverb region: log_ratio ~ prime + (1 | participant) + (1 + prime | item)

	ß	SE	df	t	*p*
Intercept	0.02201	0.12812	14.86778	0.172	0.866
Prime	0.11345	0.09583	14.92994	1.184	0.255

Including incorrect trials: Long region: log_ratio ~ prime + (1 | participant) + (1 | item)

	ß	SE	df	t	*p*
Intercept	0.07739	0.08856	15.94764	0.874	0.39518
Prime	0.16672	0.05556	897.54747	3.001	<0.01

Section 4.5.3 (Accuracy – Experiment 3)

Face verification: Answer ~ prime + (1 + prime| participant) + (1 | item)

	ß	SE	z-value	*p*
Intercept	2.7261	0.2014	13.535	<0.0001
Prime	-0.3842	0.2020	-1.902	0.0571

Sentence verification: Answer ~ prime + (1 | participant) + (1 | item)

	ß	SE	z-value	*p*
Intercept	1.58108	0.21469	7.365	<0.0001
Prime	0.04973	0.08238	0.604	0.546

Section 4.5.4 (Reaction times – Experiment 3)

Face verification: RT ~ prime*match + (1 | participant) + (1 | item)

	ß	SE	df	t	*p*
Intercept	6.990082	0.035516	65.201114	196.818	<0.0001
Prime	0.030808	0.008537	814.045317	3.609	<0.001
Match	0.058065	0.008648	818.756839	6.714	<0.0001
Prime * Match	-0.028995	0.008649	817.432855	-3.352	<0.001

Sentence verification: RT ~ prime*match + (1 + prime | participant) + (1 + prime | item)

	ß	SE	df	t	*p*
Intercept	8.5977470	0.0177931	26.1140501	483.207	<0.0001
Prime	-0.0007377	0.0031928	14.2069426	-0.231	0.821

Note: Complete model outputs can be obtained upon request.
