# Supplementary material for: Integration of Social Context vs. Linguistic Reference During Situated Language Processing
Source: Front Psychol. 2021 Aug 2;12:547360. doi: 10.3389/fpsyg.2021.547360 (PMC8365155; doi:10.3389/fpsyg.2021.547360)
Supplement: Supplementary file 2 [file Data_Sheet_2.pdf]

Supplementary Material 2

For a list of experimental sentences, scenes and primes, see Appendices A.1 (experimental sentences), B.1 and B.2 (primes), and C.2 (experimental scenes for Experiments 1 and 2) in Münster (2016, see https://pub.uni-bielefeld.de/record/2906648.).

An example of a critical scene (Figure A, item 5 b)) from Experiment 3 illustrates the changes made in comparison to the experimental scenes in Experiments 1 and 2: Compared to item 5 b) in Appendix C.2 (Münster, 2016), in Experiment 3, the positive and negative emotional facial expressions of the target agent (the cat) and the competitor agent (the rat) were enhanced and the middle character (the patient) was removed.

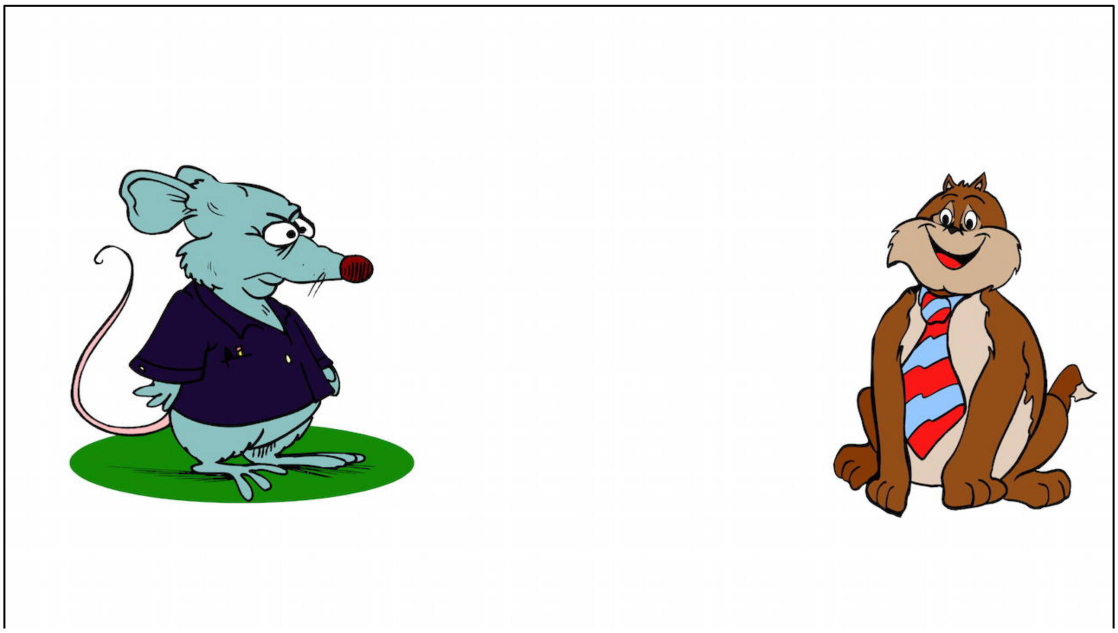

Figure A. Example of a critical scene from Experiment 3.
